# Randomised placebo-controlled trial of antenatal corticosteroids for planned birth in twins (STOPPIT-3): study protocol

**DOI:** 10.1136/bmjopen-2023-078778

**Published:** 2024-01-18

**Authors:** Sarah Murray, Jessica Thompson, Rosie C Townsend, Manuela Deidda, Kathleen Anne Boyd, Jane E Norman, John Norrie, James P Boardman, Karen Luyt, Asma Khalil, Debra Bick, Keith Reed, Jane Denton, Natasha Fenwick, Catriona Keerie, Rebecca Reynolds, Sarah Jane Stock

**Affiliations:** 1 Centre for Reproductive Health, The University of Edinburgh, Edinburgh, UK; 2 Health Economics and Health Technology Assessment, University of Glasgow, Glasgow, UK; 3 University of Nottingham Executive Office, University of Nottingham, Nottingham, UK; 4 Edinburgh Clinical Trials Unit, The University of Edinburgh, Edinburgh, UK; 5 Bristol Medical School, University of Bristol, Bristol, UK; 6 Molecular and Clinical Sciences Research Institute, St George’s University of London, London, UK; 7 Warwick Clinical Trials Unit, Warwick Medical School, University of Warwick, Coventry, UK; 8 CEO, Parent Infant Foundation, London, UK; 9 Co lead Elizabeth Bryan Multiple Births Centre, Imperial College London, London, UK; 10 Research and Resources Officer, Twins Trust, London, UK; 11 University of Edinburgh College of Medicine and Veterinary Medicine, Edinburgh, UK; 12 Centre for Cardiovascular Science, The University of Edinburgh, Edinburgh, UK; 13 Centre for Medical Informatics, The University of Edinburgh Usher Institute of Population Health Sciences and Informatics, Edinburgh, UK

**Keywords:** OBSTETRICS, NEONATOLOGY, Fetal medicine

## Abstract

**Introduction:**

The aim of the STOPPIT-3 study is to determine the clinical and cost effectiveness of antenatal corticosteroids (ACS) prior to planned birth of twins in a multicentre placebo-controlled trial with internal pilot.

**Methods and analysis:**

This study will comprise a multicentre, double-blinded, randomised, placebo-controlled trial in at least 50 UK obstetric units. The target population is 1552 women with a twin pregnancy and a planned birth between 35 and 38+6 weeks’ gestation recruited from antenatal clinics. Women will be randomised to Dexamethasone Phosphate (24 mg) or saline administered via two intramuscular injections 24 hours apart, 24–120 hours prior to scheduled birth.

**Outcomes:**

The primary outcome is need for respiratory support within 72 hours of birth. Secondary and safety outcomes will be included. Cognitive and language development at age 2 years will be assessed in a subset of participants using the Parent report of Children’s Abilities-Revised questionnaire. We will also determine the cost effectiveness of the treatment with ACS compared with placebo.

**Ethics and dissemination:**

STOPPIT-3 has been funded and approved by the National Institute of Healthcare Research. It has been approved by the West Midlands Research Ethics Committee (22/WM/0018). The results will be disseminated via publication in peer-reviewed journals and conference presentation and will also be communicated to the public via links with charity partners and social media.

**Trial sponsor:**

The University of Edinburgh and Lothian Health Board ACCORD, The Queen’s Medical Research Institute, 47 Little France Crescent, Edinburgh, EH16 4TJ.

**Trial registration number:**

ISRCTN59959611.

**STRENGTHS AND LIMITATIONS OF THIS STUDY**

- Double-blind randomised multicentre trial.
- Cost effectiveness of antenatal corticosteroids use in twin pregnancy.
- Large sample size.
- Internal pilot to assess recruitment rate and intracluster correlation coefficient.
- Long term follow-up only possible in a subset of participants within the timeframe of the trial.

## Introduction

The overall aim of STOPPIT-3 is to address the uncertainty regarding the effectiveness of antenatal corticosteroids (ACS) prior to a planned birth of twins in the late preterm and early term period. ACS are widely administered via intramuscular (IM) injection to women at risk of preterm birth (defined as birth less than 37 completed weeks gestation) to reduce morbidity and mortality in babies born too early^1^ and have been recommended since the 1990s. ACS are known to be most effective if birth occurs 24–48 hours following administration of the first dose, with little or no benefit seen if birth is 7 days or more after administration.^1^

Twin pregnancy is common and associated with adverse outcomes for the babies, accounting for ~3% of live births but ~15%–20% of all neonatal care admissions.^2^ 2019 NICE guidance for twin pregnancy recommends planned birth at 37+0 weeks gestation in uncomplicated dichorionic (DC) twins (twins that have separate placentae), and planned birth at 36+0 weeks gestation in uncomplicated monochorionic (MC) twins (twins that share a placenta (~20% of twins)).^3^ Planned birth is by induction of labour (IOL) or caesarean section (CS). These slightly earlier, non-spontaneous births are at increased risk of respiratory morbidity and needing respiratory support requiring neonatal care admission.

There is, however, currently little evidence that ACS are as effective in twins, and similarly little evidence that ACS are effective in the late preterm and early term period which is the period that NICE recommend that twins are born.^3^ Evidence as to whether women having planned birth of twins should receive ACS is both conflicting and confusing, with practice known to be highly variable across the UK in this area. ACS are widely given to women with twin pregnancies having planned birth, despite recognition that ACS *may* have adverse effects on growth and neurodevelopment.^4 5^ There is some evidence that ACS in singleton pregnancies in the late preterm period (34+0–36+6 weeks) and/or prior to planned CS at term (37+0–38+6 weeks gestation)^6^ may have short-term benefits reducing respiratory morbidity and neonatal care admission. This evidence is often extrapolated to twin pregnancy, however, there is almost no evidence showing benefits of ACS from women with twin pregnancies. Differences in the pharmacokinetics of ACS,^7^ and mechanisms of fetal maturation (which may be accelerated in twins),^8^ may mean that ACS have different effectiveness at late preterm and early term gestations.

ACS are not devoid of harm. A large RCT of ACS in late preterm singletons demonstrated an increase in neonatal hypoglycaemia in the ACS group compared with placebo (number needed to harm 11).^9^ ACS have well-recognised detrimental effects on fetal growth (birth weight, length and head circumference) and conflicting results on neurodevelopment. Three studies (one RCT follow-up and two longitudinal studies) have shown detrimental effects on neurodevelopment following ACS exposure,^4 5 10^ but a recently published prospective follow-up study of the above RCTs of ACS in late preterm singletons demonstrated no adverse effect of ACS on childhood neurodevelopment outcomes.^11^ The balance of risk and benefit needs to be determined for twin pregnancies. Reducing term (>37 weeks gestation) neonatal care admission is a UK national priority. It poses a high cost to the NHS and separation of mothers and babies is detrimental to maternal well-being, mother–infant bonding and breastfeeding.^12^ There is evidence that ACS reduce serious respiratory morbidity and neonatal unit admission but there is potential for short (eg, hypoglycaemia) and long-term harms (eg, neurodevelopment). Either currently a substantial number of babies miss a morbidity sparing treatment; or a substantial number receive a potentially harmful treatment unnecessarily as practice varies substantially across the UK. STOPPIT-3 will provide the evidence to address this uncertainty.

## Methods

### Design

STOPPIT-3 is a multicentre double-blind randomised placebo controlled trial to determine the clinical and cost effectiveness of ACS versus placebo in women with a viable twin pregnancy with planned birth between 35+0 and 38+6 weeks gestation. An internal pilot phase will take place to assess recruitment rates. A nested economics analysis will assess cost-effectiveness of ACS versus placebo. The primary objective is to test the hypothesis that ACS reduce neonatal morbidity including the need for respiratory support within 72 hours of birth. The secondary objectives are to determine the effect of ACS on severe respiratory morbidity, perinatal mortality, maternal outcomes including breastfeeding and infection and the cost-effectiveness of treatment with ACS compared with placebo. The effect of ACS compared with placebo on childhood cognitive and language development at the age of two will also be assessed in a subset of twins. The study opened for recruitment in August 2022 and recruitment will run until August 2025.

### Health technology being assessed

A single course of Dexamethasone Phosphate (24 mg) given in two divided doses by IM injection to the thigh or buttock by appropriately qualified clinical or research staff 24 hours apart (±4 hours). Two formulations of ACS, Dexamethasone and Betamethasone, are recommended in the UK. Dexamethasone has been chosen over Betamethasone as it does not need to be stored in a fridge, is cheaper and is more widely available worldwide.

### Population

The target population is women with a confirmed viable twin pregnancy and planned birth between 35+0 and 38+6 weeks gestation. Women who are booked for their delivery at one of the participating study sites and who appear to meet the study eligibility criteria will be invited to participate. Medical records of women pregnant with twins will be reviewed by the maternity care teams for individual recruitment potential into the trial. We anticipate that all eligible women expecting twins and attending for antenatal care in each of the sites will be invited to participate. Women who appear to fulfil the inclusion criteria for the trial will be approached by a member of maternity care team after confirmation of a viable twin pregnancy at an appropriate antenatal clinic or ultrasound visit, usually between 16 and 24 weeks gestation. Women will be provided with a written short trial summary at this time. Women will then be provided with a detailed patient information leaflet and consent form later in their pregnancy (between 32 and 36 weeks gestation, see online supplemental material). The timings outlined for giving women trial information should be followed if possible, however flexibility for approaching women is permitted and deviation from the timelines set out here will not be recorded as a protocol deviation. If the woman waives this opportunity for early information but still wishes to participate, consent may be taken after a shorter time interval. Where possible the reason for an eligible woman being excluded or declining participation will be recorded, for input into trial metrics as per the Consolidated Standards of Reporting Trials statement.^13^

10.1136/bmjopen-2023-078778.supp2 Supplementary data

### Eligibility criteria

The following inclusion criteria will apply at the screening assessment (all must apply):

- Aged 16 years or older and able to provide electronic or written consent.
- Viable twin pregnancy (MC or DC) with a planned birth* scheduled between 35+0 and 38+6 weeks gestation including women who have a planned birth due to logistic reasons (eg, availability of beds or staff), parental preference or other maternal or fetal indications.
- Gestation established by scan at≤16 weeks according to NICE guidelines and known chorionicity.
- ≥24 hours* and<7 days until planned birth.
  *Birth must be planned to take place at 35 or more weeks gestation, after IOL or CS. At the point of randomisation, there must be ≥24 hours until the planned CS or IOL date to allow two doses of the study drug to be administered, at 24 hours (±4 hours) apart prior to the planned birth.

The following exclusion criteria will apply:

- Unable to give informed consent.
- Known or suspected major congenital fetal anomaly at the time of inclusion (defined as any structural or chromosomal anomaly that would influence management at or around birth or in the immediate postnatal period. Suspected isolated minor anomalies with lesser medical, functional or cosmetic consequences; or isolated limb abnormalities such as talipes can be included).
- Diabetes (pre-existing or gestational)—corticosteroid use may significantly disrupt glycaemic control in women with diabetes, with potential to ‘unblind’ treatment allocation and pose risk to these women. The effect of corticosteroids prior to planned CS in women with diabetes will be examined in other studies.
- Receipt of ACS within the 7 days prior to randomisation.
- Sensitivity, contraindication or intolerance to any of the ACS or any of its excipients.
- Chorionicity or gestational age are unknown.
- Other serious pregnancy morbidities which indicate either birth before 35 weeks or urgent birth within 24 hours.

### Outcomes

#### Primary outcome

The primary outcome is need for respiratory support within 72 hours of birth. This outcome encompasses a range of levels of support consisting of one or more of the following: continuous positive airway pressure (CPAP); supplemental oxygen by high-flow nasal cannulae for at least two consecutive hours; need for supplemental oxygen by low flow nasal cannulae or incubator oxygen for at least four continuous hours; mechanical ventilation; extracorporeal membrane oxygenation (ECMO). Stillbirth or neonatal death within 72 hours of birth will be included as competing events.

#### Secondary outcomes

- Severe respiratory morbidity within 72 hours after birth (defined as one or more of the following: CPAP or high-flow nasal cannula for at least 12 continuous hours; supplemental oxygen with a fraction of inspired oxygen of at least 0.30 for at least 24 continuous hours; mechanical ventilation; ECMO; stillbirth; neonatal death within 72 hours of birth).
- Any admission to neonatal care (ie, admission for any reason and for any duration).
- Neonatal care admission within 72 hours of birth for 48 hours or more or any neonatal care admission (within 28 days of birth) or those requiring surfactant treatment or nitric oxide therapy.
- Apgar score at 5 min.
- Umbilical arterial cord pH.
- Umbilical arterial cord base excess.
- Newborn hypoglycaemia diagnosed within 48 hours of birth (defined as blood glucose of less than 2.0 mmol per litre).
- Newborn neonatal jaundice (defined as those requiring treatment with phototherapy according to NICE threshold for gestation and postnatal age).
- Birth weight centile.
- Head circumference at birth.
- All cause early onset sepsis within 72 hours of birth (defined as culture positive (pure growth from blood or CSF of a known bacterial pathogen) or culture negative (acute onset of illness with three or more predefined clinical signs)).
- Extended perinatal mortality (stillbirth or neonatal death up to 28 days).
- Stillbirth (death in utero).
- Neonatal death (death within 28 days of birth).
- Exclusive breastmilk nutrition at discharge.
- Confirmed or suspected maternal postpartum infection during hospital admission (defined by a new prescription of antibiotics, confirmed systemic infection on culture or endometritis as defined by the US Centres for Disease Control and Prevention).
- Cost effectiveness of treatment with ACS compared with placebo.
- Childhood cognitive and language development at 2 years of age determined by the Parent Report of Children’s Abilities-Revised (PARCA-R) score^14^ (in the first 340 women recruited to the trial).

### Consent and baseline assessment

After the potential participant has had adequate time to consider involvement in the study, she will be contacted by a member of the trial team to ascertain interest in the trial. The consent, baseline assessment and randomisation for STOPPIT-3 are anticipated to be combined and conducted as a single visit before the planned birth and will wherever possible coincide with routine preadmission appointments to help minimise additional visits. Written informed consent will be taken by a member of the maternity care team. Consent should be provided within 7 days of randomisation/investigational medicinal product (IMP) administration. The original signed consent form will be stored in the Investigator Site File, with a copy given to the woman and a copy added to the medical notes. The women’s demographics, medical history, obstetric history, current pregnancy information and inclusion/exclusion criteria will be collected and entered on the electronic case report form (eCRF) by a member of the trial team. The inclusion/exclusion criteria will be further assessed by a doctor (delegated by the principal investigator (PI)) and they will complete and sign the eligibility form confirming the woman meets the study criteria to participate and is suitable for randomisation. A letter will be sent to the registered general practitioner to inform them of the woman’s participation in the trial.

### Randomisation

Randomisation to ACS or placebo will be performed immediately prior to administration, 24 hours to 120 hours before the planned birth. Randomisation is performed using a web-based randomisation system managed by Edinburgh Clinical Trials Unit (ECTU) via a web portal. Users will be assigned a unique study identifier and will be required to enter minimal patient details prior to randomisation. As this is a large trial (1552 women), group imbalances are unlikely therefore a simple allocation sequence with no minimisation criteria will be used. Study participants, trial investigators and medical staff providing care will remain blinded to treatment allocation. The randomisation process will assign each participant with a study drug treatment pack number and the first dose of IMP should be given immediately following randomisation with the second dose administered 24 hours (±4 hours) after the first dose. Participants will be allocated to receive either:

1. Corticosteroid group—two doses of 12 mg dexamethasone by IM injection 24 hours (±4 hours) apart.
2. Placebo group—two doses of matching placebo (sodium chloride 0.9%) by IM injection 24 hours (±4 hours) apart.

### Data collection and management

Birth and neonatal information will be extracted from the woman’s±babies’ medical notes and information recorded in the eCRF by a member of the maternity research team. Trial data will be collected by members of the maternity care team delegated by the PI. A unique trial identifier will be allocated to each participating woman at randomisation and this unique number will be used for data collection within the trial. Identifiers will be stored in separate tables from the main data tables within the trial database and only delegated members of the team will be granted access to these tables (see Data Sharing Plan, (online supplemental material).

10.1136/bmjopen-2023-078778.supp1 Supplementary data

### Long term follow-up assessments

The first 340 STOPPIT-3 participants recruited will be asked to complete the PARCA-R questionnaire (online or paper copy) at 2 years (to assess the cognitive and language development).

### Statistical analysis and sample size

The statistical analysis will be according to the intention to treat principle (ie, all participants will remain in their allocated group for analysis). Statistical significance will be at the 5% level with corresponding 95% CI presented. Randomised groups will be described at baseline and follow-up using mean (SD), median (IQR) and counts (with percentages) as appropriate.

For the primary outcome (respiratory support within 72 hours of birth), the OR (and 95% CI) for the treatment effect of ACS will be estimated adjusting for mode of delivery, treatment centre (if appropriate) and chorionicity with logistic regression. To account for the clustering effect within twin pairs, a random-effects logistic regression model will be used by fitting pregnant woman as a random effect.

Continuous secondary outcomes will be analysed using linear regression, and binary categorical secondary outcomes will be analysed using logistic regression as per the primary outcome. Secondary outcomes with more than two categories will be analysed using multinomial logistic regression.

Subgroup analyses, for example by sex of twins, chorionicity and presence of maternal comorbidity (eg, hypertension) will be considered.

No interim analyses are planned other than re-estimation of the intraclass correlation (ICC, assumed to be 0.3) following the internal pilot. This will be done by estimating the 95% CI (without, and possibly with, adjustment for covariates) as per the event rate around the observed ICC at 200 women with complete data, and if this 95% CI does not contain 0.3 corrective action will be taken.

We plan to recruit 1552 women randomised at 1:1 to ACS or placebo prior to planned birth. We will have 90% power at the 5% significance level to detect a relative difference in the neonatal primary outcome of respiratory support within 72 hours of birth between the groups of 33% (absolute difference of 4%) assuming an event rate of 12% in the placebo group and an ICC of 0.3, assuming 1% of missing data for the primary outcome.

### Health economic analysis

The primary within trial analysis will be a cost-effectiveness analysis which will estimate the incremental cost per reduction in respiratory support (initiated within 72 hours after birth, ie, the study primary outcome), with the time horizon spanning from birth to child hospital discharge or 28 days, whichever is sooner.

The costs of the intervention will be calculated as the daily cost of ACS medication and the associated administration costs. Hospital attendances required to administer ACS will be included. The direct medical costs post birth will be calculated based on resource utilisation accruing for the care of new born (after birth respiratory treatment; admission to neonatal care) and women (type of delivery, inpatient stays; hospital transfers, etc) including adverse events. Resource utilisation for woman and child will be collected from the clinical hospital records up to 28 days post birth.

The mean cost and mean outcome associated with the intervention and the control arm will be estimated using generalised linear model, which will tackle non-normality of data, adjusting for relevant covariates (eg, type of delivery; MC/DC twins) and adjusting for women-level clustering, in line with the statistical analysis.

If evidence of differences between the treatment arms in terms of effectiveness, costs or cost-effectiveness are found in the trial, a decision analytic model will be developed to explore the cost-effectiveness of ACS administration over a medium (2 year) and longer term (lifetime) horizon. The medium term analysis will use data from the final trial follow-up period in childhood (eg, PARCA-R, any medical records available, etc), to account for costs and consequences which are associated with ACS treatment over the neonatal period (hypoglycaemia; neonatal health) and childhood (mortality; cognitive development metabolic illness).

### Internal pilot

There will be an internal pilot phase over the first 10 months of the trial when we aim to recruit 159 women and have 36 sites open. There is a clear stop/go traffic light criterion for trial progression beyond the internal pilot.

### Co-enrolment

Coenrolment in STOPPIT-3 and another non-interventional research study (eg, sample only or questionnaire studies) is permitted and this does not require any formal written documentation. This includes the related STOPPIT-3 mechanistic study (STOPPIT-M) sponsored by the NIHR Efficacy and Mechanism Evaluation (EME) programme (reference NIHR133388).

Coenrolment in STOPPIT-3 and another CTIMP or interventional non-CTIMP (eg, diagnostic, device or surgical interventions) are permitted provided an assessment on the safety of study participants, interventions involved, participant burden and the potential impact on the study endpoints have been considered. This assessment will be performed and documented in line with the Sponsor policy on coenrolment.

### Ineligible and non-recruited participants

Women who consent to participate in the study, but who spontaneously give birth or undergo IOL or CS prior to randomisation will not be eligible for randomisation. Such women who did consent to participate will be withdrawn but will remain on the eCRF system and reported in recruitment metrics as ineligible post consent. No delivery outcomes will be collected and they will continue receiving standard care under the management of a clinician, as per current guidelines. The woman’s care will not be affected due to non-trial participation. Randomisation and IMP administration should be performed contiguously to minimise the chance of spontaneous labour or delivery between randomisation and IMP administration.

### Unblinding

Breaking of the study blind will only be performed where knowledge of the treatment is essential for the clinical management of the woman or neonate. Unblinding is managed by the central Edinburgh team.

### Withdrawal of study participants

Patients are free to withdraw at any point or can be withdrawn by the investigator. The primary reason for withdrawal will be recorded in the patient’s eCRF and medical record.

### Trial management and oversight

The multisite trial will be coordinated by a Project Management Group consisting of the grant holders and the Trial Management Team within the ECTU. A Trial Steering Committee (TSC) will be established to oversee the conduct and progress of the trial. An independent data monitoring committee will be established to oversee the safety of participants in the trial.

### Patient and public involvement (PPI)

The study was designed in response to a recent global priority setting partnership of 1000 parents of twins who identified 10 research priorities for future health of multiples and their families. Two of the top 10 priorities will be addressed within STOPPIT-3: (1) How can we reduce multiples’ (the babies) admission to the NNU and can we reduce their length of stay in the NNU and (2) what are the short-term and long-term outcomes in multiple pregnancies and are these outcomes affected by antenatal events and medical interventions?

The study has been co-designed with two charities who represent parents with twins, the Twins Trust and the Elizabeth Bryan Multiple Birth Centre (formerly the Multiple Births Foundation). We consulted parents, through both charities at the grant submission stage and also at the protocol stage specifically on study design, the primary outcome and effect size, secondary outcomes and recruitment strategies.

Patients and the public are also involved in the TSC for this study with two individual patients as well as involvement of the coapplicants from the Twins Trust and the Elizabeth Bryan Multiple Birth Centre. A virtual parent advisory group has been set up to review patient facing materials and advice on dissemination plans. Individual study participants will be sent a summary of the study findings when the main study is published.

### Ethics and dissemination

The study will be conducted in accordance with the principles of the International Conference on Harmonisation Tripartite Guideline for Good Clinical Practice (ICH GCP). The results of the study, together with other mandated information, will be uploaded to the European clinical trials database within 1 year of the end of the study. Summaries of results will also be made available to investigators for dissemination within their clinics (where appropriate and according to their discretion). The results will be disseminated via publication in peer-reviewed journals and conference presentation and will also be communicated to the public via links with charity partners and social media.

## Supplementary Material

Reviewer comments

Author's manuscript
