# Supplementary material for: Randomised placebo-controlled trial of antenatal corticosteroids for planned birth in twins (STOPPIT-3): study protocol
Source: BMJ Open. 2024 Jan 18;14(1):e078778. doi: 10.1136/bmjopen-2023-078778 (PMC10806667; doi:10.1136/bmjopen-2023-078778)
Supplement: Supplementary data [file bmjopen-2023-078778supp002.pdf]

STOPPIT 3

INSERT LOCAL DETAILS HERE

STOPPIT-3 PIL and consent 27062023 V8.0 IRAS Project ID: 1004166

Participant Information Sheet

A Randomised Placebo-Controlled Trial of Antenatal Corticosteroids for Planned Birth in Twins: STOPPIT-3

You are invited to take part in a research trial. To help you decide whether or not to take part, it is important for you to understand why the research is being done and what it will involve. Please take time to read the following information carefully. Talk to others about the trial if you wish. Contact us if there is anything that is not clear, or if you would like more information. Take time to decide whether or not you wish to take part.

Why have I been invited to take part?

You have been asked to take part as you are pregnant with twins.

What is the purpose of the trial?

This trial aims to find out if the drug antenatal corticosteroids (ACS) given to women with a twin pregnancy prior to a planned birth of twins after 35 weeks of pregnancy reduces breathing difficulties in the twin babies.

Antenatal Corticosteroids (ACS) help to mature babies' lungs and may reduce breathing difficulties and the need for high levels of respiratory support. They are routinely used in singleton pregnancies which deliver early, but the use of ACS in twin births has not been studied in detail and so it is not clear if they will work in twin pregnancies. Because of the lack of evidence, there is currently no guidance on giving ACS in twin pregnancies, so whether or not women pregnant with twins receive steroids as part of routine care varies depending on their hospital. ACS may also have some unwanted side effects such as lowering babies' blood sugars, affecting babies' growth and possibly affecting the babies' brain development. We need to be certain about the benefits and risks of giving ACS before all women with twin pregnancy in the UK are offered a course of ACS prior to a planned birth.

Twin pregnancies are monitored more closely as they have a higher risk of complications than a singleton pregnancy, and there is a greater chance of the babies being born before 37 weeks of pregnancy. Twin births account for about 3% of live births but around 15-20% of admissions to the neonatal unit.

Current guidance recommends that twins who share a placenta (monochorionic twins) should be born from 36 weeks of pregnancy if there are no medical problems requiring earlier birth, whilst twins with a placenta each (dichorionic twins) should be born from 37 weeks of pregnancy, as evidence shows this is safer than delivering later on in the pregnancy. Being born slightly early means that twins are at higher risk of admission to neonatal units for support with their breathing, which separates mothers and babies at a crucial time.

FUNDED BY NIHR | National Institute for Health Research

This study is funded by the National Institute for Health Research (NIHR) Health Technology Assessment (HTA) programme (NIHR131352). The views expressed are those of the author(s) and not necessarily those of the NIHR or the Department of Health and Social Care.

We are performing this trial in NHS Centres throughout the UK. Women with a twin pregnancy who have a planned birth after 35 weeks of pregnancy are invited to participate in the trial. Women who agree to take part in the trial will be treated with either ACS or a placebo (dummy drug). A placebo, or dummy drug, is an inactive substance which seems to be a "real medical treatment". In this trial the placebo is a sodium chloride solution which will look identical to the ACS injections. These will be administered by injection prior to the planned birth.

We will compare the two groups to see if there are differences in the need for extra healthcare support after birth. If we find that the use of ACS improves health in twin babies, it could be used in the NHS straight away.

We need around 1,550 women to participate in the trial to be able to see if ACS works in twins.

Do I have to take part?

No, it is up to you to decide whether or not to take part. If you do decide to take part, you will be given this information sheet to keep and be asked to sign a consent form. If you decide to take part, you are still free to withdraw at any time and without giving a reason. Deciding not to take part or withdrawing from the trial will not affect the healthcare that you receive, or your legal rights.

What will happen if I take part?

An outline of the trial is given below. Where possible we will combine any additional visits needed for the trial with your routine antenatal appointments to avoid too many extra appointments However, combining the trial visit with routine visits may mean a longer visit overall. Some of the assessments may be carried out virtually before the visit using a remote secure system.

Giving consent to take part

The maternity care team will review your maternity notes and determine whether you are eligible to take part in the trial. If you are eligible, you will be asked if you would like to participate by a member of the maternity team, and this will usually be during one of your routine antenatal appointments. If you have verbally agreed to participate you will be invited to attend hospital 24 -120 hours (1-5 days) hours prior to admission for planned birth, when the ACS or placebo will be administered. You will be asked to sign a consent form before the trial drug is administered, and you will be given a copy of the signed consent form to keep for your records.

If you consented to take part but your baby is born before 35 weeks' gestation, or you are induced before your planned delivery date, you will not be eligible to proceed in the trial. Whether or not you receive ACS in this case will be decided by your doctor on an individual basis.

Trial data collection

If you consent to take part, a member of the research team will collect some information about you, including:

- medical history, including current medication
- obstetric history (previous pregnancies/births)

FUNDED BY NIHR | National Institute for Health Research

This study is funded by the National Institute for Health Research (NIHR) Health Technology Assessment (HTA) programme (NIHR131352). The views expressed are those of the author(s) and not necessarily those of the NIHR or the Department of Health and Social Care.

- current pregnancy information

This will be entered on to the trial database by a member of the research team.

Randomisation

Sometimes we don't know which treatment is best. To find out, we need to make comparisons between different treatments. We do this by putting people into groups and giving each group a different treatment; the results are then compared to see if one is better. To try to make sure the groups are the same to start with, each participant is put into a group by chance (randomly). This is called randomisation. The results are then compared.

In STOPPIT-3 you will be randomised to one of two groups. There is approximately a 50:50 chance that you will be randomised into either group.

1. Corticosteroid Group: Two separate doses of ACS (Dexamethasone) by intramuscular injection (either to the thigh or buttock)

OR

Placebo Group: Two separate doses of visually matching placebo (Sodium Chloride, also known as saline) by intramuscular injection (either to the thigh or buttock).

The trial is a double blind trial, and so neither you nor your doctor/medical team will know which treatment group you are in (although, if your doctor needs to find out s/he can do so). Everyone involved in the trial - women, medical professionals caring for women and trial investigators - will remain blinded to treatment allocation until the trial is completed.

You will be given a study card with contact information in case you have any questions. This card should be used if clinical staff need to know which treatment you received in an emergency. This process is called 'unblinding' and the treatment information needed can be obtained by the clinical team and the central trial team who will use the approved study database to obtain the information required. You should carry this study card with you while in the trial.

Your GP will be informed, by letter, of your participation in STOPPIT-3.

We hope to establish if there are any effects of ACS for the mothers. Women have told us the outcomes that are important to them, and so we will be looking at rates of infection after birth and any impact on breastfeeding. We will also look at any complications in babies. Information about the health of you and your baby in the period after birth will be taken from your medical records. We will collect data from you/your babies' medical record until discharge or 28 days postnatal (whichever is sooner).

We will ask parents to complete a questionnaire (online or by post if you prefer) called a PARCA-R (Parent Report of Children's Abilities-Revised). This questionnaire will be used to assess children's development. This will be done when your babies are 2 years old, which is when the questionnaire is designed to be used and is a good time to get an indication of any long-term positive effects of ACS on babies' health. Before sending the questionnaire to you we will ask the maternity care team to check that the details you have given us are still up to date.

If any new information about the drug we are studying becomes available during the course of the trial, all women will be informed by their preferred method of communication.

FUNDED BY NIHR | National Institute for Health Research

This study is funded by the National Institute for Health Research (NIHR) Health Technology Assessment (HTA) programme (NIHR131352). The views expressed are those of the author(s) and not necessarily those of the NIHR or the Department of Health and Social Care.

If you decide to take part in STOPPIT-3 you may still be eligible to take part in other research studies involving medicines and this will be checked by the STOPPIT-3 team. You will also be able to take part in other types of research, for example studies where you are asked to complete a questionnaire, or studies that are looking at the data collected when you are treated in hospital.

What are the possible benefits of taking part?

We don't know if you and your baby will directly benefit from taking part in this trial. Information we obtain from your participation in the study may help inform on the future healthcare of other patients. Taking part will help create much needed evidence on the use of ACS prior to a planned birth of twins, which will help women and babies in future.

What are the possible disadvantages of taking part?

You may be required to spend some extra time at a routine antenatal visit. If you do choose to take part in the study you may also be required to spend extra time at the hospital when you attend the planned pre-birth appointment. This is because we will need to record extra information specifically for the trial, for example, it will take time to discuss the trial with you, and to take consent to participate. This is additional to what is normally discussed at antenatal appointments and so will take a little extra time.

Where possible any additional visits required for the trial will be combined with routine antenatal appointments. Therefore you will not receive any recompense for taking part in the trial; this includes things like travel expenses. However you will receive a £20 high street shopping voucher for completing the follow-up questionnaire as recognition for your time and input.

There are very few recognised immediate side effects of a short course of ACS as used in this study. ACS are routinely used in pregnancy when women are at risk of preterm birth with very low rates of side effects. Allergic reactions to ACS are extremely rare. Headaches and short-term sleep disturbance have been reported after ACS but not confirmed.

What if there are any problems?

If you have a concern about any aspect of this trial please contact <insert name and contact details here> who will do their best to answer your questions.

In the unlikely event that something goes wrong, and you are affected during the research and this is due to someone's negligence, then you may have grounds for a legal action for compensation against your local hospital or the trial sponsors: University of Edinburgh/NHS Lothian, but you may have to pay your legal costs. The normal National Health Service complaints mechanisms will still be available to you (if appropriate).

What will happen if I don't want to carry on with the trial?

You can withdraw from the trial at any point, without giving a reason; this would not affect your clinical care. We will keep information about you that we already have.

FUNDED BY NIHR | National Institute for Health Research

This study is funded by the National Institute for Health Research (NIHR) Health Technology Assessment (HTA) programme (NIHR131352). The views expressed are those of the author(s) and not necessarily those of the NIHR or the Department of Health and Social Care.

STOPPIT3

INSERT LOCAL DETAILS HERE

What happens when the trial is finished?

We will write a clinical trial report, which may be used for publication and presentation at scientific meetings. All information in this report will be anonymised. These results will be uploaded to a publically accessible database within a year of the trial ending.

All trial data will be kept for at least 25 years from the end of the trial.

Will my taking part be kept confidential?

All the information we collect during the course of the research will be kept confidential and there are strict laws which safeguard your privacy at every stage.

How will we use information about you?

We will need to use information from you and your babies from your hospital notes for this research project.

Any details we have about you will be kept securely, with access restricted on a secure bespoke database managed by the University of Edinburgh Clinical Trials Unit (ECTU). This information will be used to contact you about the trial by doctors or researchers running this trial. With your consent we will collect the following personal information:

- Name, ethnicity, Dates of Birth and Hospital number (NHS or CHI) for both you and your babies. The NHS number or Community Health Index number (in Scotland) is used for health care purposes and it uniquely identifies a person.
- Address (postal and email) and contact details: this is so we can continue to follow up your babies and contact you at the end of the trial with information about the results.
- If you are asked (and agree) to sign the consent form electronically the IP address from the computer you use to sign the form will be collected

Personal information collected will be retained (with your consent) for use in future studies into the long term outcomes of ACS. It is necessary to keep personal information for you and your babies to link trial data (treatment group) to long term outcomes (NHS records/school records). Any future studies would require separate governance approvals.

We will inform your GP that you are taking part, with your consent.

All the information we collect about you and your baby will be stored in a secure database and only the trial researchers will have access to this. When you are randomised, you will be allocated a unique trial number and individuals that do not need to know who you are will see only your trial number and not your personal information. All the information we collect during the course of the research will be kept confidential and there are strict laws which safeguard your privacy at every stage.

FUNDED BY NIHR | National Institute for Health Research

This study is funded by the National Institute for Health Research (NIHR) Health Technology Assessment (HTA) programme (NIHR131352). The views expressed are those of the author(s) and not necessarily those of the NIHR or the Department of Health and Social Care.

INSERT LOCAL DETAILS HERE

We will keep all information about you safe and secure, and will not share any personal information held about you with any other organisation. However, individuals from the trial funder, regulatory authority or Sponsor organisation may review trial information and sections of you/your babies' medical notes to ensure that the trial is being done properly.

Once we have finished the trial, we will keep some of the data so we can check the results. We will write our reports in such a way that no-one can work out that you took part in the trial.

What are your choices about how your information is used?

You can stop being part of the trial at any time, without giving a reason, but we will keep the information about you that we already have. It is important that we keep the data collected up until the point at which you withdraw as it documents the care that you received and therefore forms part of your maternity care record.

If you choose to stop taking part in the trial, we will ask you if we can continue collecting information about your health and your babies' health from your hospital notes. If you do not want this to happen, tell us and we will stop. We will discuss this with you but if you decide not to participate further the research team will collect this information from your notes unless you tell them (or another member of the clinical team) that you do not agree to this. We think this is important to collect this information, so that we can find out if giving ACS is beneficial.

We need to manage your records in specific ways for the research to be reliable. This means that we won't be able to let you see or change the data we hold about you. Research could go wrong if data is removed or changed.

Future research studies organised by other Universities may also investigate more about the effects of ACS in twins. With your permission, we would like to share *anonymised* information collected during this trial with other researchers running similar studies in the future. If you do not want your anonymised information to be shared with other organisations for future research you must make this clear in the attached consent form

Some studies may ask to use data that identifies you. If this is the case, a member of the trial team from Edinburgh University will contact you to discuss this and request permission. Identifiable data will be never be shared with another organisation without your consent.

Where can you find out more about how your information is used?
You can find out more about how we use your information here:
www.hra.nhs.uk/information-about-patients/

- our leaflet available from **www.hra.nhs.uk/patientdataandresearch**
- by asking one of the research team
- by sending an email to STOPPIT.Trial@ed.ac.uk or the Administrator: Lorraine Adamson, email: L.D.Adamson@ed.ac.uk

What will happen to the results of the trial?

The results of the trial will be published in research journals and presented at scientific meetings.

FUNDED BY NIHR | National Institute for Health Research

This study is funded by the National Institute for Health Research (NIHR) Health Technology Assessment (HTA) programme (NIHR131352). The views expressed are those of the author(s) and not necessarily those of the NIHR or the Department of Health and Social Care.

We will also update you (the participant) with a summary of the trial findings through our trial website and other social media platforms. We do not expect the trial results to be available until early 2026, and so we will update the website and social media sites with trial progress. All information used for trial updates and final results will be anonymous, and it will not be possible to identify individuals from any published material.

Who is organising and funding the research?

This trial is organised and sponsored by the University of Edinburgh and NHS Lothian.

The trial is being funded by the National Institute for Health Research (NIHR), Health Technology Assessment (HTA) Programme (Project: 131352). The views and opinions are those of the authors and do not necessarily reflect those of the HTA programme, NIHR or the Department of Health.

Who has reviewed the trial?

This trial has been reviewed and approved by the following bodies:

(1) Research Ethics Committee (REC). All research in the NHS is looked at by an independent group of people called a Research Ethics Committee. REC approval was obtained on 14/02/2022 (ref 22/WM/0018).

(2) Medicines and Healthcare Regulatory Agency (MHRA). The MHRA review and authorise all clinical research studies investigating the safety or efficacy of a drug. The MHRA approval was obtained on 15/02/2022 (ref CTA 01384/0268/001-0001).

(3) NHS Management Approval. Each hospital that takes part in a clinical trial must also review and approve the trial before their patients can be approached to take part. NHS Management Approval was obtained from (site name) on XX (ref)

(4) User groups/stakeholders. The trial protocol, information sheets and trial design have also been reviewed by relevant user groups and stakeholders. The Twins Trust and Multiple Births Foundation have provided input to the trial materials, and advised on various aspects of the management of the trial.

Researcher Contact Details

If you have any further questions about the trial, please contact <insert name> on <insert phone number> or email on: <insert email address>.

Independent Contact Details

If you would like to discuss this trial with someone independent of the trial, please contact TBC <insert contact details>.

Complaints

If you wish to make a complaint about the trial please contact:

Adapt depending on research site <insert contact details>

For NHS Lothian this is:
Patient Experience Team
2 – 4 Waterloo Place, Edinburgh, EH1 3EG
feedback@nhslothian.scot.nhs.uk Tel: 0131 536 3370

FUNDED BY NIHR | National Institute for Health Research

This study is funded by the National Institute for Health Research (NIHR) Health Technology Assessment (HTA) programme (NIHR131352). The views expressed are those of the author(s) and not necessarily those of the NIHR or the Department of Health and Social Care.

STOPPIT 3

INSERT LOCAL DETAILS HERE

STOPPIT-3 PIL and consent 27062023 V8.0 IRAS Project ID: 1004166

CONSENT FORM

A Randomised Placebo-Controlled Trial of Antenatal Corticosteroids for Planned Birth in Twins:
STOPPIT-3

Please **initial** box

1. I confirm that I have read and understand the information sheet (STOPPIT-3 PIL Version 8.0, dated 27/06/2023) for the above trial. I have had the opportunity to consider the information, ask questions and have had these questions answered satisfactorily. ☐

2. I understand that my participation is voluntary and that I am free to withdraw at any time, without giving any reason and without my medical care and/or legal rights being affected. ☐

3. I give permission for the research team to access my and my babies medical records for the purposes of this research trial. ☐

4. I understand that relevant sections of my, and my babies', medical notes and data collected during the trial including my personal details may be looked at by individuals from the regulatory authorities and from the Sponsor(s) (NHS Lothian and the University of Edinburgh), from other NHS Boards or Trusts involved in the trial, where it is relevant to my taking part in this research. I give permission for these individuals to have access to my records. ☐

5. I give permission for my and my babies personal information (including name, address, date of birth, telephone number and consent form) to be passed to the University of Edinburgh Trials Unit for administration of the trial. ☐

6. I give permission for my Community Health Index (CHI) number/hospital number or NHS number to be collected and passed to the University of Edinburgh Clinical Trials Unit ☐

7. I agree to my General Practitioner being informed of my participation in the trial. ☐

8. I understand that data collected about me and my babies (related to the trial and personal) during the trial will be kept for 25 years, and might be contacted in the future about related research studies. ☐

9. I agree to my anonymised data being used in future studies. Yes ☐ No ☐

10. I understand that the information held and maintained by [enter site/hospital name] may be used to help contact me or provide information about my health status ☐

11. I agree to take part in the above trial. ☐

Name of Person Giving Consent	Date	Signature
Name of Person Receiving Consent	Date	Signature

1x original – into Site File; 1x copy – to Participant; 1x copy – into medical record

FUNDED BY NIHR | National Institute for Health Research

This study is funded by the National Institute for Health Research (NIHR) Health Technology Assessment (HTA) programme (NIHR131352). The views expressed are those of the author(s) and not necessarily those of the NIHR or the Department of Health and Social Care.
