# Supplementary material for: Randomised placebo-controlled trial of antenatal corticosteroids for planned birth in twins (STOPPIT-3): study protocol
Source: BMJ Open. 2024 Jan 18;14(1):e078778. doi: 10.1136/bmjopen-2023-078778 (PMC10806667; doi:10.1136/bmjopen-2023-078778)
Supplement: Supplementary data [file bmjopen-2023-078778supp001.pdf]

Academic and Clinical Central Office for Research and Development

The STOPPIT Study Data Sharing Plan

STOPPIT 3

STOPPIT-M

**Study Title**	STOPPIT-3: A Randomised Placebo-Controlled Trial of Antenatal Corticosteroids for Planned Birth in Twins:<br><br>STOPPIT-M: Infant hypothalamic-pituitary-adrenal axis responses following antenatal corticosteroids and perinatal outcomes: a mechanism of action of health intervention study
**Chief Investigator**	STOPPIT-3: Professor Sarah Stock, Co-CI Dr. Sarah Murray<br>STOPPIT-M: Professor Rebecca Reynolds
**ISRCTN Number**	59959611
**Sponsor**	University of Edinburgh & NHS Lothian
**Version**	v1.0 11 October 2023

Table of Contents

1 Introduction 2
2 Data type 2
2.1 Type of scientific data expected to be generated in the trial 2
2.2 Dataset responsibility 2
2.3 Other associated documentation 3
2.3.1 Protocol 3
2.3.2 Statistical Analysis Plan / Health Economics Analysis Plan 3

Murray S, *et al. BMJ Open* 2024; 14:e078778. doi: 10.1136/bmjopen-2023-078778

2.3.3 Publication material 3
3 Data preservation, access, and timelines 3
3.1 Where will scientific data be archived 3
3.2 Archiving timelines 3
3.3 Data Access 3
3.3.1 Application Type 3
3.3.2 Application Process 4
4 Access, Distribution, or Reuse Considerations 4
4.1 Factors affecting subsequent access 4
4.2 What form will the sharable dataset take 4
4.3 Restrictions on data sharing 4
4.4 Protections for privacy, rights, and confidentiality of human research participants 4
4.5 Process of de-identification/anonymisation of the data 4
5 Oversight of Data Management and Sharing 4
6 Acknowledgement in output 4
7 References 4

1 Introduction

The STOPPIT 3 and STOPPIT M study data is held within Edinburgh Clinical Trials Unit (ECTU). The STOPPIT 3 data sharing plan therefore aligns with the ECTU Central Office SOP ECTU_OP_15: Data Access Request and Application Management SOP (Version 2.0; 11 Oct 2021) **[1].**

This data sharing plan is also in line with the STOPPIT 3 & STOPPIT M Publication policy **[2]**.

This data sharing plan has been approved by the Chief Investigator and the STOPPIT-3 trial statistician in Edinburgh Clinical Trials Unit (ECTU).

2 Data type

2.1 Type of scientific data expected to be generated in the trial

The data to be shared includes both meta data including the study protocol, case report forms and data dictionaries, and research participant data.

2.2 Dataset responsibility

The study team are responsible for the sharing of datasets arising from the STOPPIT 3 & STOPPIT M study. The Sponsor (University of Edinburgh and NHS Lothian) are joint data controllers for the study.

Murray S, *et al. BMJ Open* 2024; 14:e078778. doi: 10.1136/bmjopen-2023-078778

2.3 Other associated documentation

2.3.1 Protocol

The STOPPIT 3 & STOPPIT M study was registered on the ISRCTN clinical trials registry (https://www.isrctn.com/ISRCTNISRCTN599596111) before the participant recruitment commenced. The protocol will also be published in an open access journal.

2.3.2 Statistical Analysis Plan / Health Economics Analysis Plan

The Statistical Analysis Plan (SAP) will be available on request.

The Health Economics Analysis Plan (HEAP) will also be available on request.

2.3.3 Publication material

The STOPPIT 3 and STOPPIT M publication and dissemination policy describes how trial outputs will be managed, reviewed and disseminated. This policy follows ICMJE criteria for authorship. It outlines the requirements for research outputs from the study and any additional requirements for reporting and disseminating results. It is expected that study findings will be published as soon as possible, in a peer-reviewed open access journal or platform. The final report for NIHR HTA Journals Library will be submitted within 24 months of the end of the study (as defined in the protocol).

3 Data preservation, access, and timelines

3.1 Where will scientific data be archived

Once the study is closed and the statistical analysis has been completed, all study data will be stored on a suitable secure server in the Edinburgh Clinical Trials Unit. Study data will be available on request from the ECTU data sharing team (see section 3.3).

3.2 Archiving timelines

Study data and metadata will be kept for a minimum of 25 years from the protocol defined end of study point. When the minimum retention period has elapsed, study documentation will not be destroyed without permission from the sponsor.

3.3 Data Access

3.3.1 Application Type

Applications for access to study data which are received from an external source, out with ECTU, are subject to review by the ECTU Data Sharing team. There are two categories of data access request. A Data Access Request Application Type A is made when the study is currently recruiting participants, closed to recruitment with participants in follow-up or when the study is closed (all recruitment and follow-up completed) but the main statistical analysis is not yet complete. This should be made using form Data Access Application Form Type A (OP-F02).

A Data Access Request Application Type B is made when the study is closed, and the statistical analysis has been completed. This should be made using form Data Access Application Form Type B (OP-F03)

3.3.2 Application Process

In the first instance, email requests for study data should be made via email to: ECTUdatashare@ed.ac.uk.

Further details about this process are available in ECTU Central Office SOP ECTU_OP_15 **[1].**

4 Access, Distribution, or Reuse Considerations

4.1 Factors affecting subsequent access

If the application is approved, the review panel will also consider the method of access and whether any additional agreements will be required prior to the access being granted. It may be necessary to further consult with external colleagues (e.g. contracts) at this stage.

4.2 What form will the sharable dataset take

The sharable dataset will include the statistical analysis dataset and the health economic analysis dataset. In general, we will only share the statistical analysis dataset and the health economics data set, but consideration will be given to sharing source dataset tables upon request.

4.3 Restrictions on data sharing

If the study results have not yet been published, it may be appropriate to embargo any data access requests until post-publication to ensure the results are not undermined.

4.4 Protections for privacy, rights, and confidentiality of human research participants

All shared data will be de-identified prior to release and in accordance with permissions listed in the STOPPIT 3 & STOPPIT M protocol, ethical approvals, and Patient Information Sheet Consent Form (PISCF)

4.5 Process of de-identification/anonymisation of the data

Suitably qualified personnel will de-identify the data prior to release, if his has not already been done.

5 Oversight of Data Management and Sharing

ECTU will retain oversight of data management, and of sharing processes for requests that come to ECTU, involving data held by ECTU.

6 Acknowledgement in output

Secondary users of data should follow the STOPPIT 3 and STOPPIT M Publication policy for acknowledgments and authorship **[2]**.

7 References

Murray S, *et al. BMJ Open* 2024; 14:e078778. doi: 10.1136/bmjopen-2023-078778

1. ECTU Central Office SOP ECTU_OP_15: Data Access Request and Application Management SOP; Version 2.0; 11 Oct 2021; available at https://www.ed.ac.uk/sites/default/files/atoms/files/ectu_sop_op_15_data_access_request_and_application_management_v2.0.pdf (accessed 11th November 2022).

2. STOPPIT-3 & M Publication Policy; Version 1.0; 17 Jan 2022; available from the STOPPIT trial management team at stoppit.trial@ed.ac.uk

Murray S, *et al. BMJ Open* 2024; 14:e078778. doi: 10.1136/bmjopen-2023-078778
