# Supplementary material for: Randomised placebo-controlled trial of antenatal corticosteroids for planned birth in twins (STOPPIT-3): study protocol
Source: BMJ Open. 2024 Jan 18;14(1):e078778. doi: 10.1136/bmjopen-2023-078778 (PMC10806667; doi:10.1136/bmjopen-2023-078778)
Supplement: Author's manuscript [file bmjopen-2023-078778.draft_revisions.pdf]

BMJ Open is committed to open peer review. As part of this commitment we make the peer review history of every article we publish publicly available.

When an article is published we post the peer reviewers' comments and the authors' responses online. We also post the versions of the paper that were used during peer review. These are the versions that the peer review comments apply to.

The versions of the paper that follow are the versions that were submitted during the peer review process. They are not the versions of record or the final published versions. They should not be cited or distributed as the published version of this manuscript.

BMJ Open is an open access journal and the full, final, typeset and author-corrected version of record of the manuscript is available on our site with no access controls, subscription charges or pay-per-view fees (http://bmjopen.bmj.com).

If you have any questions on BMJ Open's open peer review process please email info.bmjopen@bmj.com

BMJ Open

Study protocol: a randomised placebo-controlled trial of antenatal corticosteroids for planned birth in twins (STOPPIT-3)

Journal:	*BMJ Open*
Manuscript ID	bmjopen-2023-078778
Article Type:	Protocol
Date Submitted by the Author:	11-Aug-2023
Complete List of Authors:	Murray, Sarah; The University of Edinburgh, MRC Centre for Reproductive Health<br>Thompson, Jessica ; The University of Edinburgh, MRC Centre for Reproductive Health<br>Townsend, Rosie; The University of Edinburgh, MRC Centre for Reproductive Health<br>Deidda, Manuela; University of Glasgow, Health Economics and Health Technology assessment<br>Boyd, Kathleen; University of Glasgow, Health Economics & Health Technology Assessment<br>Norman, Jane; University of Nottingham, University of Nottingham Executive Office<br>Norrie, John; The University of Edinburgh, Edinburgh Clinical Trials Unit<br>Boardman, James; The University of Edinburgh, MRC Centre for Reproductive Health<br>Luyt, Karen; University of Bristol, Bristol Medical School<br>Khalil, Asma; St George's University of London, Molecular and clinical sciences research institute<br>Bick, Debra; University of Warwick, Warwick Clinical Trials Unit, Warwick Medical School<br>Reed, Keith; Parent infant foundation , CEO<br>Denton, Jane; Imperial College London, Co lead Elizabeth Bryan Multiple Births Centre<br>Fenwick, Natasha; Twins Trust, Research and Resources Officer<br>Keerie, Catriona; University of Edinburgh College of Medicine and Veterinary Medicine<br>Reynolds, Rebecca; The University of Edinburgh, Centre for Cardiovascular Science<br>Stock, Sarah; The University of Edinburgh Usher Institute of Population Health Sciences and Informatics, Centre for Medical Informatics; Public Health Scotland,
Keywords:	OBSTETRICS, NEONATOLOGY, Fetal medicine < OBSTETRICS

SCHOLARONE™
Manuscripts

Study protocol: a randomised placebo-controlled trial of antenatal corticosteroids for planned birth in twins (STOPPIT-3)

Authors:

Sarah R Murray*, Jess Thompson*, Rosie C Townsend, Manuela Deidda, Kathleen Boyd, Jane E Norman, John Norrie, James P Boardman, Karen Luyt, Asma Khalil, Debra Bick, Keith Reed, Jane Denton, Natasha Fenwick, Catriona Keerie, Rebecca M Reynolds, Sarah J Stock

*joint first author

Correspondence to Dr Sarah Rose Murray; sarah.murray@ed.ac.uk

Sarah R Murray University of Edinburgh MRC centre for Reproductive Health, Edinburgh UK. Sarah.murray@ed.ac.uk

Jessica Thompson University of Edinburgh MRC centre for Reproductive Health, Edinburgh UK. Jessica.thompson@ed.ac.uk

Rosemary C Townsend University of Edinburgh Usher Institute, Edinburgh, UK. Rtownse2@exseed.ed.ac.uk

Manuela Deidda University of Glasgow Institute of Health and Wellbeing, Glasgow, UK. Manuela.deidda@glasgow.ac.uk

Kathleen Boyd University of Glasgow Institute of Health and Wellbeing, Glasgow, Uk.

Kathleen.boyd@glasgow.ac.uk

Jane E Norman University of Nottingham Executive Office, Nottingham, UK. Jane.norman@nottingham.ac.uk

John Norrie University of Edinburgh Clinical Trials Unit, Edinburgh, UK. J.norrie@ed.ac.uk

James P Boardman University of Edinburgh MRC Centre for Reproductive Health, Edinburgh, UK. James.boardman@ed.ac.uk

Karen Luyt, University of Bristol Translational Health Sciences, Bristol, UK. Karen.luyt@bristol.ac.uk

For peer review only

Asma Khalil, St George's University of London, London, UK. Asma.khalil@stgeorges.nhs.uk.

Debra Bick, University of Warwick Clinical Trials Unit, Warwick, UK. Debra.bick@warwick.ac.uk

Keith Reed CEO Parent-Infant Foundation, keith@parentinfantfoundation.org.uk

Jane Denton, Elizabeth Bryan Multiple Births Centre, Imperial College Health Care NHS Trust, London, UK. Jane.denton1@nhs.net

Natasha Fenwick, Research and Resources Officer, Twins Trust, natashafenwick@twinstrust.org

Catriona Keerie University of Edinburgh Clinical Trials Unit, Edinburgh, UK. Catriona.keerie@ed.ac.uk

Rebecca M Reynolds University of Edinburgh Centre for Cardiovascular Science, Edinburgh, UK. R.reynolds@ed.ac.uk

Sarah J Stock University of Edinburgh Usher Institute, Edinburgh, UK. Sarah.stock@ed.ac.uk

For peer review only

Abstract:

Introduction The aim of the STOPPIT-3 study is to determine the clinical and cost effectiveness of antenatal corticosteroids prior to planned birth of twins in a multi-centre placebo-controlled trial with internal pilot.

Methods and analysis This study will comprise a multicentre, double-blinded, randomised, placebo-controlled trial in at least 50 UK Obstetric units. The target population is 1552 women with a twin pregnancy and a planned birth between 35 and 38+6 weeks' gestation recruited from antenatal clinics. Women will be randomised to Dexamethasone Phosphate (24mg) or saline administered via two intramusuclar injections 24 hours apart, 24-120 hours prior to scheduled birth. Outcomes: The primary outcome is need for respiratory support within 72 hours of birth. Secondary and safety outcomes will be included. Cognitive and language development at age two years will be assessed in a subset of participants using the Parent report of Children's Abilities- Revised [PARCA-R] questionnaire. We will also determine the cost effectiveness of the treatment with ACS compared to placebo.

For peer review only - http://bmjopen.bmj.com/site/about/guidelines.xhtml

Ethics and dissemination STOPPIT-3 has been funded and approved by the National Institute of Healthcare Research. It has been approved by the West Midlands Research Ethics Committee (22/WM/0018). The results will be disseminated via publication in peer-reviewed journals and conference presentation and will also be communicated to the public via links with charity partners and social media.

Abstract word count: 221

Article Summary

Version Protocol V.7.0, Date 24 March 2023

Trial Registration Number ISRCTN59959611

Strengths and limitations of this study

- Double blind randomised multicentre trial
- Cost effectiveness of ACS use in twin pregnancy
- Large sample size
- Internal pilot to assess recruitment rate and intra-cluster correlation co-efficient (ICC)
- Long term follow up only possible in a subset of participants within the timeframe of the trial

Manuscript Word Count: 3775

Introduction

The overall aim of STOPPIT-3 is to address the uncertainty regarding the effectiveness of Antenatal Corticosteroids (ACS) prior to a planned birth of twins in the late preterm and early term period. ACS are widely administered via intramuscular injection to women at risk of preterm birth (defined as birth less than 37 completed weeks gestation) to reduce morbidity and mortality in babies born too early (1) and have been recommended since the 1990s. ACS are known to be most effective if birth

For peer review only

occurs 24 to 48 hours following administration of the first dose, with little or no benefit seen if birth is seven days or more after administration (1).

Twin pregnancy is common and associated with adverse outcomes for the babies, accounting for ~3% of live births but ~15-20% of all neonatal care admissions (2). 2019 NICE guidance for twin pregnancy recommends planned birth at 37+0 weeks gestation in uncomplicated dichorionic (DC) twins (twins that have separate placentae), and planned birth at 36+0 weeks gestation in uncomplicated monochorionic (MC) twins (twins that share a placenta [~20% of twins])(3). Planned birth is by induction of labour (IOL) or caesarean section (CS). These slightly earlier, non-spontaneous births are at increased risk of respiratory morbidity and needing respiratory support requiring neonatal care admission.

There is, however, currently little evidence that ACS are as effective in twins, and similarly little evidence that ACS are effective in the late preterm and early term period which is the period that NICE recommend that twins are born (3). Evidence as to whether women having planned birth of twins should receive ACS is both conflicting and confusing, with practice known to be highly variable across the United Kingdom in this area. ACS are widely given to women with twin pregnancies having planned birth, despite recognition that ACS *may* have adverse effects on growth and neurodevelopment (4, 5). There is some evidence that ACS in singleton pregnancies in the late preterm period (34+0 – 36+6 weeks) and/or prior to planned CS at term (37+0 - 38+6 weeks gestation)(6), may have short term benefits reducing respiratory morbidity and neonatal care admission. This evidence is often extrapolated to twin pregnancy, however, there is almost no evidence showing benefits of ACS from women with twin pregnancies. Differences in the pharmacokinetics of ACS (7), and mechanisms of fetal maturation (which may be accelerated in twins)(8), may mean that ACS have different effectiveness at late preterm and early term gestations.

ACS are not devoid of harm. A large RCT of ACS in late preterm singletons demonstrated an increase in neonatal hypoglycaemia in the ACS group compared to placebo (number needed to harm 11)(9). ACS have well recognised detrimental effects on fetal growth (birthweight, length and head circumference) and conflicting results on neurodevelopment. Three studies (one RCT follow up and two longitudinal studies) have shown detrimental effects on neurodevelopment following ACS exposure (4, 5, 10) but a recently published prospective follow up study of the above RCTs of ACS in late preterm singletons demonstrated no adverse effect of ACS on childhood neurodevelopment

For peer review only - http://bmjopen.bmj.com/site/about/guidelines.xhtml

outcomes (14). The balance of risk and benefit needs to be determined for twin pregnancies. Reducing term (>37 weeks gestation) neonatal care admission is a UK national priority. It poses a high cost to the NHS and separation of mothers and babies is detrimental to maternal wellbeing, mother-infant bonding and breastfeeding (11). There is evidence that ACS reduce serious respiratory morbidity and neonatal unit admission but there is potential for short (e.g. hypoglycaemia) and long-term harms (e.g. neurodevelopment). Either currently a substantial number of babies miss a morbidity sparing treatment; or a substantial number receive a potentially harmful treatment unnecessarily as practice varies substantially across the UK. STOPPIT-3 will provide the evidence to address this uncertainty.

Methods

Design STOPPIT-3 is a multicentre double blind randomised placebo controlled trial to determine the clinical and cost effectiveness of ACS versus placebo in women with a viable twin pregnancy with planned birth between 35+0 and 38+6 weeks gestation. An internal pilot phase will take place to assess recruitment rates. A nested economics analysis will assess cost-effectiveness of ACS versus placebo. The primary objective is to test the hypothesis that ACS reduce neonatal morbidity including the need for respiratory support within 72 hours of birth. The secondary objectives are to determine the effect of ACS on severe respiratory morbidity, perinatal mortality, maternal outcomes including breastfeeding and infection and the cost-effectiveness of treatment with ACS compared to placebo. The effect of ACS compared to placebo on childhood cognitive and language development at the age of two will also be assessed in a subset of twins.

Health technology being assessed A single course of Dexamethasone Phosphate (24mg) given in two divided doses by intra-muscular (IM) injection to the thigh or buttock by appropriately qualified clinical or research staff 24 hours apart (+/- 4 hours). Two formulations of ACS, Dexamethasone and Betamethasone, are recommended in the UK. Dexamethasone has been chosen over Betamethasone as it does not need to be stored in a fridge, is cheaper and is more widely available Worldwide.

Population The target population is women with a confirmed viable twin pregnancy and planned birth between 35+0-38+6 weeks gestation. Women who are booked for their delivery at one of the participating study sites and who appear to meet the study eligibility criteria will be invited to participate. Medical records of women pregnant with twins will be reviewed by the maternity care

For peer review only - http://bmjopen.bmj.com/site/about/guidelines.xhtml

teams for individual recruitment potential into the trial. We anticipate that all eligible women expecting twins and attending for antenatal care in each of the sites will be invited to participate. Women who appear to fulfil the inclusion criteria for the trial will be approached by a member of maternity care team after confirmation of a viable twin pregnancy at an appropriate antenatal clinic or ultrasound visit, usually between 16-24 weeks gestation. Women will be provided with a written short trial summary at this time. Women will then be provided with a detailed patient information leaflet and consent form later in their pregnancy (between 32-36 weeks gestation). The timings outlined for giving women trial information should be followed if possible, however flexibility for approaching women is permitted and deviation from the timelines set out here will not be recorded as a protocol deviation. If the woman waives this opportunity for early information but still wishes to participate, consent may be taken after a shorter time interval. Where possible the reason for an eligible woman being excluded or declining participation will be recorded, for input into trial metrics as per the CONSORT statement. (12)

Eligibility criteria The following inclusion criteria will apply at the screening assessment (all must apply):

Aged 16 years or older and able to provide electronic or written consent

Viable twin pregnancy (monochorionic or dichorionic) with a planned birth* scheduled between 35+0 and 38+6 weeks gestation including women who have a planned birth due to logistic reasons (e.g. availability of beds or staff), parental preference or other maternal or fetal indications.

Gestation established by scan at ≤16 weeks according to NICE guidelines and known chorionicity

≥ 24 hours* and < 7 days until planned birth

*Birth must be planned to take place at 35 or more weeks gestation, after induction of labour (IOL) or CS. At the point of randomisation there must be ≥ 24 hours until the planned CS or IOL date to allow two doses of the study drug to be administered, at 24 hours (+/- 4 hours) apart prior to the planned birth.

For peer review only - http://bmjopen.bmj.com/site/about/guidelines.xhtml

The following exclusion criteria will apply:

Unable to give informed consent

Known or suspected major congenital fetal anomaly at the time of inclusion (defined as any structural or chromosomal anomaly that would influence management at or around birth or in the immediate postnatal period. Suspected isolated minor anomalies with lesser medical, functional or cosmetic consequences; or isolated limb abnormalities such as talipes can be included).

Diabetes (pre-existing or gestational) - Corticosteroid use may significantly disrupt glycaemic control in women with diabetes, with potential to 'unblind' treatment allocation and pose risk to these women. The effect of corticosteroids prior to planned CS in women with diabetes will be examined in other studies.

Receipt of ACS within the seven days prior to randomisation

Sensitivity, contraindication or intolerance to any of the ACS or any of its excipients

Chorionicity or gestational age are unknown

Other serious pregnancy morbidities which indicate either birth before 35 weeks or urgent birth within 24 hours

For peer review only

Outcomes

Primary outcome The primary outcome is need for respiratory support within 72 hours of birth. This outcome encompasses a range of levels of support consisting of one or more of the following: continuous positive airway pressure (CPAP); supplemental oxygen by high-flow nasal cannulae for at least 2 consecutive hours; need for supplemental oxygen by low flow nasal cannulae or incubator oxygen for at least 4 continuous hours; mechanical ventilation; Extracorporeal Membrane Oxygenation (ECMO). Stillbirth or Neonatal death within 72 hours of birth will be included as competing events.

For peer review only - http://bmjopen.bmj.com/site/about/guidelines.xhtml

Secondary outcomes

Severe respiratory morbidity within 72 hours after birth (defined as one or more of the following: CPAP or high-flow nasal cannula for at least 12 continuous hours; Supplemental oxygen with a fraction of inspired oxygen of at least 0.30 for at least 24 continuous hours; Mechanical ventilation; ECMO; Stillbirth; Neonatal death within 72 hours of birth)

Any admission to neonatal care (i.e. admission for any reason and for any duration)

Neonatal care admission within 72 hours of birth for 48 hours or more or any Neonatal care admission (within 28 days of birth) or those requiring surfactant treatment or nitric oxide therapy

Apgar score at 5 minutes

Umbilical arterial cord pH

Umbilical arterial cord base excess

Newborn hypoglycaemia diagnosed within 48 hours of birth (defined as blood glucose of less than 2.0 mmol per litre).

Newborn neonatal jaundice (defined as those requiring treatment with phototherapy according to NICE threshold for gestation and postnatal age)

Birthweight centile

Head circumference at birth

All cause early onset sepsis within 72 hours of birth (defined as culture positive [pure growth from blood or CSF of a known bacterial pathogen] or culture negative [acute onset of illness with 3 or more predefined clinical signs])

Extended perinatal mortality (stillbirth or neonatal death up to 28 days)

Stillbirth (death *in utero*)

Neonatal death (death within 28 days of birth)

For peer review only

For peer review only - http://bmjopen.bmj.com/site/about/guidelines.xhtml

Exclusive breastmilk nutrition at discharge

Confirmed or suspected maternal postpartum infection during hospital admission (defined by a new prescription of antibiotics, confirmed systemic infection on culture, or endometritis as defined by the US Centers for Disease Control and Prevention)

Cost effectiveness of treatment with ACS compared to placebo

Childhood cognitive and language development at two years of age determined by the Parent Report of Children's Abilities-Revised (PARCA-R) score (13) (in the first 340 women recruited to the trial)

Consent and Baseline assessment

After the potential participant has had adequate time to consider involvement in the study, she will be contacted by a member of the trial team to ascertain interest in the trial. The consent, baseline assessment and randomisation for STOPPIT-3 are anticipated to be combined and conducted as a single visit before the planned birth and will wherever possible coincide with routine pre-admission appointments to help minimise additional visits. Written informed consent will be taken by a member of the maternity care team. Consent should be provided within 7 days of randomisation/IMP administration. The original signed consent form will be stored in the Investigator Site File, with a copy given to the woman and a copy added to the medical notes. The women's demographics, medical history, obstetric history, current pregnancy information and inclusion /exclusion criteria will be collected and entered on the eCRF by a member of the trial team. The inclusion/ exclusion criteria will be further assessed by a doctor (delegated by the PI) and they will complete and sign the eligibility form confirming the woman meets the study criteria to participate and is suitable for randomisation. A letter will be sent to the registered GP to inform them of the woman's participation in the trial.

Randomisation

Randomisation to ACS or placebo will be performed immediately prior to administration, 24 hours to 120 hours before the planned birth. Randomisation is performed using a web-based randomisation system managed by ECTU via a web portal. Users will be assigned a unique study identifier and will be required to enter minimal patient details prior to randomisation. As this is a large trial (1552 women), group imbalances are unlikely therefore a simple allocation sequence with no minimisation criteria will be used. Study participants, trial investigators and medical staff providing care will remain

For peer review only - http://bmjopen.bmj.com/site/about/guidelines.xhtml

blinded to treatment allocation. The randomisation process will assign each participant with a study drug treatment pack number and the first dose of IMP should be given immediately following randomisation with the second dose administered 24 hours (+/- 4 hours) after the first dose. Participants will be allocated to receive either:

1. Corticosteroid group – two doses of 12mg dexamethasone by IM injection 24 hours (+/- 4 hours) apart.

2. Placebo group – two doses of matching placebo (sodium chloride 0.9%) by IM injection 24 hours (+/- 4 hours) apart.

Data collection and management

Birth and neonatal information will be extracted from the woman's +/-babies' medical notes and information recorded in the eCRF by a member of the maternity research team. Trial data will be collected by members of the maternity care team delegated by the PI. A unique trial identifier will be allocated to each participating woman at randomisation and this unique number will be used for data collection within the trial. Identifiers will be stored in separate tables from the main data tables within the trial database and only delegated members of the team will be granted access to these tables.

Long term Follow up Assessments

The first 340 STOPPIT-3 participants recruited will be asked to complete the PARCA-R questionnaire (on-line or paper copy) at 2 years (to assess the cognitive and language development).

Statistical analysis and sample size

The statistical analysis will be according to the intention to treat principle (i.e. all participants will remain in their allocated group for analysis). Statistical significance will be at the 5% level with corresponding 95% confidence intervals (CI) presented. Randomised groups will be described at baseline and follow-up using mean (SD), median (IQR) and counts (with percentages) as appropriate.

For the primary outcome (respiratory support within 72 hours of birth) the odds ratio (and 95% CI) for the treatment effect of ACS will be estimated adjusting for mode of delivery, treatment centre (if appropriate) and chorionicity with logistic regression. To account for the clustering effect within twin

For peer review only

For peer review only - http://bmjopen.bmj.com/site/about/guidelines.xhtml

pairs, a random effects logistic regression model will be used by fitting pregnant woman as a random effect.

Continuous secondary outcomes will be analysed using linear regression, and binary categorical secondary outcomes will be analysed using logistic regression as per the primary outcome. Secondary outcomes with more than two categories will be analysed using multinomial logistic regression.

Subgroup analyses, for example by sex of twins, chorionicity and presence of maternal co-morbidity (e.g. hypertension) will be considered.

No interim analyses are planned other than re-estimation of the intraclass correlation (ICC, assumed to be 0.3) following the internal pilot. This will be done by estimating the 95% CI (without, and possibly with, adjustment for covariates) as per the event rate around the observed ICC at 200 women with complete data, and if this 95% CI does not contain 0.3 corrective action will be taken.

We plan to recruit 1552 women randomised at 1:1 to ACS or placebo prior to planned birth. We will have 90% power at the 5% significance level to detect a relative difference in the neonatal primary outcome of respiratory support within 72 hours of birth between the groups of 33% (absolute difference of 4%) assuming an event rate of 12% in the placebo group and an ICC of 0.3, assuming 1% of missing data for the primary outcome.

Health economic analysis

The primary within trial analysis will be a cost-effectiveness analysis (CEA) which will estimate the incremental cost per reduction in respiratory support (initiated within 72 hours after birth, i.e. the study primary outcome), with the time horizon spanning from birth to child hospital discharge or 28 days, whichever is sooner.

The costs of the intervention will be calculated as the daily cost of ACS medication and the associated administration costs. Hospital attendances required to administer ACS will be included. The direct medical costs post birth will be calculated based on resource utilisation accruing for the care of new born (after birth respiratory treatment; admission to neonatal care) and women (type of delivery, inpatient stays; hospital transfers etc.) including adverse events. Resource utilisation for woman and child will be collected from the clinical hospital records up to 28 days post birth.

For peer review only - http://bmjopen.bmj.com/site/about/guidelines.xhtml

The mean cost and mean outcome associated with the intervention and the control arm will be estimated using generalised linear model (GLM), which will tackle non-normality of data, adjusting for relevant covariates (e.g. type of delivery; monochorionic/dichorionic twins), and adjusting for women-level clustering, in line with the statistical analysis.

If evidence of differences between the treatment arms in terms of effectiveness, costs or cost-effectiveness are found in the trial, a decision analytic model will be developed to explore the cost-effectiveness of ACS administration over a medium (2 year) and longer term (lifetime) horizon. The medium term analysis will utilise data from the final trial follow-up period in childhood (e.g. PARCA-R, any medical records available etc), to account for costs and consequences which are associated with ACS treatment over the neonatal period (hypoglycaemia; neonatal health) and childhood (mortality; cognitive development metabolic illness).

Internal Pilot

There will be an internal pilot phase over the first 10 months of the trial when we aim to recruit 159 women and have 36 sites open. There is a clear stop/go traffic light criterion for trial progression beyond the internal pilot.

Co-enrolment

Co-enrolment in STOPPIT-3 and another non-interventional research study (for example, sample only or questionnaire studies) is permitted and this does not require any formal written documentation. This includes the related STOPPIT-3 mechanistic study (STOPPIT-M) sponsored by the NIHR Efficacy and Mechanism Evaluation (EME) programme (reference NIHR133388).

Co-enrolment in STOPPIT-3 and another CTIMP or interventional non-CTIMP (for example, diagnostic, device or surgical interventions) are permitted provided an assessment on the safety of study participants, interventions involved, participant burden and the potential impact on the study endpoints have been considered. This assessment will be performed and documented in line with the Sponsor policy on co-enrolment.

Ineligible and non-recruited participants

For peer review only - http://bmjopen.bmj.com/site/about/guidelines.xhtml

Women who consent to participate in the study, but who spontaneously give birth or undergo IOL or CS prior to randomisation will not be eligible for randomisation. Such women who did consent to participate will be withdrawn but will remain on the eCRF system and reported in recruitment metrics as ineligible post consent. No delivery outcomes will be collected and they will continue receiving standard care under the management of a clinician, as per current guidelines. The woman's care will not be affected due to non-trial participation. Randomisation and IMP administration should be performed contiguously to minimise the chance of spontaneous labour or delivery between randomisation and IMP administration.

Unblinding

Breaking of the study blind will only be performed where knowledge of the treatment is essential for the clinical management of the woman or neonate. Unblinding is managed by the central Edinburgh team.

Withdrawal of study participants

Patients are free to withdraw at any point or can be withdrawn by the investigator. The primary reason for withdrawal will be recorded in the patient's eCRF and medical record.

Trial management and oversight

The multi-site trial will be coordinated by a Project Management Group consisting of the grant holders and the Trial Management Team within the Edinburgh Clinical Trials Unit (ECTU). A Trial Steering Committee (TSC) will be established to oversee the conduct and progress of the trial. An independent data monitoring committee (DMC) will be established to oversee the safety of participants in the trial.

Patient and Public Involvement (PPI)

The study was designed in response to a recent Global priority setting partnership of 1000 parents of twins who identified ten research priorities for future health of multiples and their families. Two of the top ten priorities will be addressed within STOPPIT-3 (i) How can we reduce multiples' (the babies) admission to the NNU and can we reduce their length of stay in the NNU and (ii) what are the

For peer review only - http://bmjopen.bmj.com/site/about/guidelines.xhtml
For peer review only

short and long-term outcomes in multiple pregnancies and are these outcomes affected by antenatal events and medical interventions?

The study has been co-designed with two charities who represent parents with twins, the Twins Trust and the Elizabeth Bryan Multiple Birth Centre (formerly the Multiple Births Foundation). We consulted parents, through both charities at the grant submission stage and also at the protocol stage specifically on study design, the primary outcome and effect size, secondary outcomes and recruitment strategies.

Patients and the public are also involved in the TSC for this study with two individual patients as well as involvement of the co-applicants from the Twins Trust and the Elizabeth Bryan Multiple Birth Centre. A virtual parent advisory group (PAG) has been set up to review patient facing materials and advise on dissemination plans. Individual study participants will be sent a summary of the study findings when the main study is published.

Ethics and dissemination

The study will be conducted in accordance with the principles of the International Conference on Harmonisation Tripartite Guideline for Good Clinical Practice (ICH GCP). The results of the study, together with other mandated information, will be uploaded to the European clinical trials database within 1 year of the end of the study. Summaries of results will also be made available to Investigators for dissemination within their clinics (where appropriate and according to their discretion). The results will be disseminated via publication in peer-reviewed journals and conference presentation and will also be communicated to the public via links with charity partners and social media.

Footnotes

Authors' contributions: SRM, SJS, MD, KB, JEN, JN, RMR, JPB, KL, AK, DB, KR, NF and JD developed the protocol. SRM, JT, RCT and SJS drafted the protocol. MD, KB, JEN, JN, RMR, JPB, KL, AK, DB, KR, NF, CK, and JD reviewed and commented on the protocol. SRM and JT contributed equally to the writing of this paper.

For peer review only - http://bmjopen.bmj.com/site/about/guidelines.xhtml
For peer review only

Funding Statement: This work was supported by the National Institute of Healthcare Research Health Technology Assessment grant number: NIHR131352

Competing interests: nil

Ethics approval: West Midlands - Edgbaston Research Ethics Committee REC reference: 22/WM/0018

Peer review: This study was extensively peer reviewed as part of the process of gaining grant funding from the NIHR HTA.

For peer review only

References

1. Roberts D, Dalziel S. Antenatal corticosteroids for accelerating fetal lung maturation for women at risk of preterm birth. Cochrane Database Syst Rev. 2006(3):Cd004454.

2. Harrison W, Goodman D. Epidemiologic Trends in Neonatal Intensive Care, 2007-2012. JAMA pediatrics. 2015;169(9):855-62.

3. National Institute for Health and Care Excellence. Twin and triplet pregnancy. 2019;NICE guideline NG137.

4. Stutchfield PR, Whitaker R, Gliddon AE, Hobson L, Kotecha S, Doull IJM. Behavioural, educational and respiratory outcomes of antenatal betamethasone for term caesarean section (ASTECS trial). Archives of disease in childhood Fetal and neonatal edition. 2013;98(3):F195-200.

5. Räikkönen K, Gissler M, Kajantie E. Associations Between Maternal Antenatal Corticosteroid Treatment and Mental and Behavioral Disorders in Children. JAMA : the journal of the American Medical Association. 2020;323(19):1924-33.

6. Saccone G, Berghella V. Antenatal corticosteroids for maturity of term or near term fetuses: systematic review and meta-analysis of randomized controlled trials. BMJ (Online). 2016;355:i5044-i.

7. Kemp MW, Newnham JP, Challis JG, Jobe AH, Stock SJ. The clinical use of corticosteroids in pregnancy. Human reproduction update. 2016;22(2):240-59.

For peer review only - http://bmjopen.bmj.com/site/about/guidelines.xhtml

8. Murray SR, Stock SJ, Cowan S, Cooper ES, Norman JE. Spontaneous preterm birth prevention in multiple pregnancy. Obstet Gynaecol, 2018;20 (1): 57-63.

9. Gyamfi-Bannerman C, Thom EA, Blackwell SC, Tita ATN, Reddy UM, Saade GR, et al. Antenatal Betamethasone for Women at Risk for Late Preterm Delivery. The New England journal of medicine. 2016;374(14):1311-20.

10. Rodriguez A, Wang Y, Khan AA, Cartwright R, Gissler M, Järvelin M-R. Antenatal corticosteroid therapy (ACT) and size at birth: A population-based analysis using the Finnish medical birth register. PLoS medicine. 2019;16(2):e1002746-e.

11. Improvement N. Reducing harm leading to avoidable admission of full-term babies to neonatal units. London: NHS Improvement. 2017.

12. Schulz KF, Altman DG, Moher D. CONSORT 2010 Statement: updated guidelines for reporting parallel group randomised trials. BMJ. 2010;340:c332.

13. Johnson S BV, Linsell L, Brocklehurst P, Marlow N, Wolke D, Manktelow B. Parent Report of Children's Abilities – Revised (PARCA-R). Technical and Interpretive Manual The University of Leicester2019 [Available from: https://www2.le.ac.uk/partnership/parca-r/questionnaire).

14 Gyamfi-Bannerman. Neurodevelopmental outcomes after late preterm antenatal corticosteroids: the alps follow-up study. AJOG. 2023 S764-S765.

For peer review only

For peer review only - http://bmjopen.bmj.com/site/about/guidelines.xhtml

Reporting checklist for protocol of a clinical trial.

Based on the SPIRIT guidelines.

Instructions to authors

Complete this checklist by entering the page numbers from your manuscript where readers will find each of the items listed below.

Your article may not currently address all the items on the checklist. Please modify your text to include the missing information. If you are certain that an item does not apply, please write "n/a" and provide a short explanation.

Upload your completed checklist as an extra file when you submit to a journal.

In your methods section, say that you used the SPIRITreporting guidelines, and cite them as:

Chan A-W, Tetzlaff JM, Gøtzsche PC, Altman DG, Mann H, Berlin J, Dickersin K, Hróbjartsson A, Schulz KF, Parulekar WR, Krleža-Jerić K, Laupacis A, Moher D. SPIRIT 2013 Explanation and Elaboration: Guidance for protocols of clinical trials. BMJ. 2013;346:e7586

		Reporting Item	Page Number
**Administrative information**			
Title	#1	Descriptive title identifying the study design, population, interventions, and, if applicable, trial acronym	1
Trial registration	#2a	Trial identifier and registry name. If not yet registered, name of intended registry	3
Trial registration: data set	#2b	All items from the World Health Organization Trial Registration Data Set	N/A
Protocol version	#3	Date and version identifier	3
Funding	#4	Sources and types of financial, material, and other support	3
Roles and responsibilities: contributorship	#5a	Names, affiliations, and roles of protocol contributors	1/2
Roles and responsibilities: sponsor contact information	#5b	Name and contact information for the trial sponsor	N/A
Roles and responsibilities: sponsor and funder	#5c	Role of study sponsor and funders, if any, in study design; collection, management, analysis, and interpretation of data; writing of the report; and the decision to submit the report for publication, including whether they will have ultimate authority over any of these activities	N/A
Roles and responsibilities: committees	#5d	Composition, roles, and responsibilities of the coordinating centre, steering committee, endpoint adjudication committee, data management team, and	13

For peer review only - http://bmjopen.bmj.com/site/about/guidelines.xhtml

		other individuals or groups overseeing the trial, if applicable (see Item 21a for data monitoring committee)	
**Introduction**			
Background and rationale	#6a	Description of research question and justification for undertaking the trial, including summary of relevant studies (published and unpublished) examining benefits and harms for each intervention	3/4
Background and rationale: choice of comparators	#6b	Explanation for choice of comparators	3/4
Objectives	#7	Specific objectives or hypotheses	5
Trial design	#8	Description of trial design including type of trial (eg, parallel group, crossover, factorial, single group), allocation ratio, and framework (eg, superiority, equivalence, non-inferiority, exploratory)	5
**Methods: Participants, interventions, and outcomes**			
Study setting	#9	Description of study settings (eg, community clinic, academic hospital) and list of countries where data will be collected. Reference to where list of study sites can be obtained	5
Eligibility criteria	#10	Inclusion and exclusion criteria for participants. If applicable, eligibility criteria for study centres and individuals who will perform the interventions (eg, surgeons, psychotherapists)	6/7
Interventions: description	#11a	Interventions for each group with sufficient detail to allow replication, including how and when they will be administered	5
Interventions: modifications	#11b	Criteria for discontinuing or modifying allocated interventions for a given trial participant (eg, drug dose change in response to harms, participant request, or improving / worsening disease)	12/13
Interventions: adherance	#11c	Strategies to improve adherence to intervention protocols, and any procedures for monitoring adherence (eg, drug tablet return; laboratory tests)	13
Interventions: concomitant care	#11d	Relevant concomitant care and interventions that are permitted or prohibited during the trial	12
Outcomes	#12	Primary, secondary, and other outcomes, including the specific measurement variable (eg, systolic blood pressure), analysis metric (eg, change from baseline, final value, time to event), method of aggregation (eg, median, proportion), and time point for each outcome. Explanation of the clinical relevance of chosen efficacy and harm outcomes is strongly recommended	7/8

For peer review only - http://bmjopen.bmj.com/site/about/guidelines.xhtml

Participant timeline	#13	Time schedule of enrolment, interventions (including any run-ins and washouts), assessments, and visits for participants. A schematic diagram is highly recommended (see Figure)	9/10
Sample size	#14	Estimated number of participants needed to achieve study objectives and how it was determined, including clinical and statistical assumptions supporting any sample size calculations	10/11
Recruitment	#15	Strategies for achieving adequate participant enrolment to reach target sample size	12/13
**Methods: Assignment of interventions (for controlled trials)**			
Allocation: sequence generation	#16a	Method of generating the allocation sequence (eg, computer-generated random numbers), and list of any factors for stratification. To reduce predictability of a random sequence, details of any planned restriction (eg, blocking) should be provided in a separate document that is unavailable to those who enrol participants or assign interventions	9
Allocation concealment mechanism	#16b	Mechanism of implementing the allocation sequence (eg, central telephone; sequentially numbered, opaque, sealed envelopes), describing any steps to conceal the sequence until interventions are assigned	9
Allocation: implementation	#16c	Who will generate the allocation sequence, who will enrol participants, and who will assign participants to interventions	9
Blinding (masking)	#17a	Who will be blinded after assignment to interventions (eg, trial participants, care providers, outcome assessors, data analysts), and how	9
Blinding (masking): emergency unblinding	#17b	If blinded, circumstances under which unblinding is permissible, and procedure for revealing a participant's allocated intervention during the trial	13
**Methods: Data collection, management, and analysis**			
Data collection plan	#18a	Plans for assessment and collection of outcome, baseline, and other trial data, including any related processes to promote data quality (eg, duplicate measurements, training of assessors) and a description of study instruments (eg, questionnaires, laboratory tests) along with their reliability and validity, if known. Reference to where data collection forms can be found, if not in the protocol	9/10

For peer review only

Data collection plan: retention	#18b	Plans to promote participant retention and complete follow-up, including list of any outcome data to be collected for participants who discontinue or deviate from intervention protocols	10
Data management	#19	Plans for data entry, coding, security, and storage, including any related processes to promote data quality (eg, double data entry; range checks for data values). Reference to where details of data management procedures can be found, if not in the protocol	10
Statistics: outcomes	#20a	Statistical methods for analysing primary and secondary outcomes. Reference to where other details of the statistical analysis plan can be found, if not in the protocol	10
Statistics: additional analyses	#20b	Methods for any additional analyses (eg, subgroup and adjusted analyses)	10/11
Statistics: analysis population and missing data	#20c	Definition of analysis population relating to protocol non-adherence (eg, as randomised analysis), and any statistical methods to handle missing data (eg, multiple imputation)	10/11
**Methods: Monitoring**			
Data monitoring: formal committee	#21a	Composition of data monitoring committee (DMC); summary of its role and reporting structure; statement of whether it is independent from the sponsor and competing interests; and reference to where further details about its charter can be found, if not in the protocol. Alternatively, an explanation of why a DMC is not needed	13
Data monitoring: interim analysis	#21b	Description of any interim analyses and stopping guidelines, including who will have access to these interim results and make the final decision to terminate the trial	13
Harms	#22	Plans for collecting, assessing, reporting, and managing solicited and spontaneously reported adverse events and other unintended effects of trial interventions or trial conduct	13
Auditing	#23	Frequency and procedures for auditing trial conduct, if any, and whether the process will be independent from investigators and the sponsor	13
**Ethics and dissemination**			
Research ethics approval	#24	Plans for seeking research ethics committee / institutional review board (REC / IRB) approval	14
Protocol amendments	#25	Plans for communicating important protocol modifications (eg, changes to eligibility criteria, outcomes, analyses) to relevant parties (eg,	14

For peer review only

For peer review only - http://bmjopen.bmj.com/site/about/guidelines.xhtml

		investigators, REC / IRBs, trial participants, trial registries, journals, regulators)	
Consent or assent	#26a	Who will obtain informed consent or assent from potential trial participants or authorised surrogates, and how (see Item 32)	9
Consent or assent: ancillary studies	#26b	Additional consent provisions for collection and use of participant data and biological specimens in ancillary studies, if applicable	9
Confidentiality	#27	How personal information about potential and enrolled participants will be collected, shared, and maintained in order to protect confidentiality before, during, and after the trial	9
Declaration of interests	#28	Financial and other competing interests for principal investigators for the overall trial and each study site	15
Data access	#29	Statement of who will have access to the final trial dataset, and disclosure of contractual agreements that limit such access for investigators	N/A
Ancillary and post trial care	#30	Provisions, if any, for ancillary and post-trial care, and for compensation to those who suffer harm from trial participation	N/A
Dissemination policy: trial results	#31a	Plans for investigators and sponsor to communicate trial results to participants, healthcare professionals, the public, and other relevant groups (eg, via publication, reporting in results databases, or other data sharing arrangements), including any publication restrictions	14
Dissemination policy: authorship	#31b	Authorship eligibility guidelines and any intended use of professional writers	14
Dissemination policy: reproducible research	#31c	Plans, if any, for granting public access to the full protocol, participant-level dataset, and statistical code	n/a
**Appendices**			
Informed consent materials	#32	Model consent form and other related documentation given to participants and authorised surrogates	n/a
Biological specimens	#33	Plans for collection, laboratory evaluation, and storage of biological specimens for genetic or molecular analysis in the current trial and for future use in ancillary studies, if applicable	n/a

The SPIRIT Explanation and Elaboration paper is distributed under the terms of the Creative Commons Attribution License CC-BY-NC. This checklist was completed on 03. February 2023 using https://www.goodreports.org/, a tool made by the EQUATOR Network in collaboration with Penelope.ai

For peer review only - http://bmjopen.bmj.com/site/about/guidelines.xhtml

BMJ Open

Study protocol: a randomised placebo-controlled trial of antenatal corticosteroids for planned birth in twins (STOPPIT-3)

Journal:	*BMJ Open*
Manuscript ID	bmjopen-2023-078778.R1
Article Type:	Protocol
Date Submitted by the Author:	19-Oct-2023
Complete List of Authors:	Murray, Sarah; The University of Edinburgh, MRC Centre for Reproductive Health<br>Thompson, Jessica ; The University of Edinburgh, MRC Centre for Reproductive Health<br>Townsend, Rosie; The University of Edinburgh, MRC Centre for Reproductive Health<br>Deidda, Manuela; University of Glasgow, Health Economics and Health Technology assessment<br>Boyd, Kathleen; University of Glasgow, Health Economics & Health Technology Assessment<br>Norman, Jane; University of Nottingham, University of Nottingham Executive Office<br>Norrie, John; The University of Edinburgh, Edinburgh Clinical Trials Unit<br>Boardman, James; The University of Edinburgh, MRC Centre for Reproductive Health<br>Luyt, Karen; University of Bristol, Bristol Medical School<br>Khalil, Asma; St George's University of London, Molecular and clinical sciences research institute<br>Bick, Debra; University of Warwick, Warwick Clinical Trials Unit, Warwick Medical School<br>Reed, Keith; Parent infant foundation , CEO<br>Denton, Jane; Imperial College London, Co lead Elizabeth Bryan Multiple Births Centre<br>Fenwick, Natasha; Twins Trust, Research and Resources Officer<br>Keerie, Catriona; University of Edinburgh College of Medicine and Veterinary Medicine<br>Reynolds, Rebecca; The University of Edinburgh, Centre for Cardiovascular Science<br>Stock, Sarah; The University of Edinburgh Usher Institute of Population Health Sciences and Informatics, Centre for Medical Informatics; Public Health Scotland,
**Primary Subject Heading**:	Obstetrics and gynaecology
Secondary Subject Heading:	Paediatrics, Research methods, Reproductive medicine
Keywords:	OBSTETRICS, NEONATOLOGY, Fetal medicine < OBSTETRICS

SCHOLARONE™
Manuscripts

Study protocol: a randomised placebo-controlled trial of antenatal corticosteroids for planned birth in twins (STOPPIT-3)

Authors:

Sarah R Murray*, Jess Thompson*, Rosie C Townsend, Manuela Deidda, Kathleen Boyd, Jane E Norman, John Norrie, James P Boardman, Karen Luyt, Asma Khalil, Debra Bick, Keith Reed, Jane Denton, Natasha Fenwick, Catriona Keerie, Rebecca M Reynolds, Sarah J Stock

*joint first author

Correspondence to Dr Sarah Rose Murray; sarah.murray@ed.ac.uk

Sarah R Murray University of Edinburgh MRC centre for Reproductive Health, Edinburgh UK. Sarah.murray@ed.ac.uk

Jessica Thompson University of Edinburgh MRC centre for Reproductive Health, Edinburgh UK. Jessica.thompson@ed.ac.uk

Rosemary C Townsend University of Edinburgh Usher Institute, Edinburgh, UK. Rtownse2@exseed.ed.ac.uk

Manuela Deidda University of Glasgow Institute of Health and Wellbeing, Glasgow, UK. Manuela.deidda@glasgow.ac.uk

Kathleen Boyd University of Glasgow Institute of Health and Wellbeing, Glasgow, Uk.

Kathleen.boyd@glasgow.ac.uk

Jane E Norman University of Nottingham Executive Office, Nottingham, UK. Jane.norman@nottingham.ac.uk

John Norrie University of Edinburgh Clinical Trials Unit, Edinburgh, UK. J.norrie@ed.ac.uk

James P Boardman University of Edinburgh MRC Centre for Reproductive Health, Edinburgh, UK. James.boardman@ed.ac.uk

Karen Luyt, University of Bristol Translational Health Sciences, Bristol, UK. Karen.luyt@bristol.ac.uk

For peer review only - http://bmjopen.bmj.com/site/about/guidelines.xhtml

Asma Khalil, St George's University of London, London, UK. Asma.khalil@stgeorges.nhs.uk.

Debra Bick, University of Warwick Clinical Trials Unit, Warwick, UK. Debra.bick@warwick.ac.uk

Keith Reed CEO Parent-Infant Foundation, keith@parentinfantfoundation.org.uk

Jane Denton, Elizabeth Bryan Multiple Births Centre, Imperial College Health Care NHS Trust, London, UK. Jane.denton1@nhs.net

Natasha Fenwick, Research and Resources Officer, Twins Trust, natashafenwick@twinstrust.org

Catriona Keerie University of Edinburgh Clinical Trials Unit, Edinburgh, UK. Catriona.keerie@ed.ac.uk

Rebecca M Reynolds University of Edinburgh Centre for Cardiovascular Science, Edinburgh, UK. R.reynolds@ed.ac.uk

Sarah J Stock University of Edinburgh Usher Institute, Edinburgh, UK. Sarah.stock@ed.ac.uk

Abstract:

Introduction The aim of the STOPPIT-3 study is to determine the clinical and cost effectiveness of antenatal corticosteroids prior to planned birth of twins in a multi-centre placebo-controlled trial with internal pilot.

Methods and analysis This study will comprise a multicentre, double-blinded, randomised, placebo-controlled trial in at least 50 UK Obstetric units. The target population is 1552 women with a twin pregnancy and a planned birth between 35 and 38+6 weeks' gestation recruited from antenatal clinics. Women will be randomised to Dexamethasone Phosphate (24mg) or saline administered via two intramusuclar injections 24 hours apart, 24-120 hours prior to scheduled birth. Outcomes: The primary outcome is need for respiratory support within 72 hours of birth. Secondary and safety outcomes will be included. Cognitive and language development at age two years will be assessed in a subset of participants using the Parent report of Children's Abilities- Revised [PARCA-R] questionnaire. We will also determine the cost effectiveness of the treatment with ACS compared to placebo.

For peer review only - http://bmjopen.bmj.com/site/about/guidelines.xhtml

Ethics and dissemination STOPPIT-3 has been funded and approved by the National Institute of Healthcare Research. It has been approved by the West Midlands Research Ethics Committee (22/WM/0018). The results will be disseminated via publication in peer-reviewed journals and conference presentation and will also be communicated to the public via links with charity partners and social media.

Abstract word count: 221

Article Summary

Version Protocol V.7.0, Date 24 March 2023

Trial Registration Number ISRCTN59959611

Trial Sponsor The University of Edinburgh & Lothian Health Board ACCORD, The Queen's Medical Research Institute, 47 Little France Crescent, Edinburgh, EH16 4TJ

Strengths and limitations of this study

- Double blind randomised multicentre trial
- Cost effectiveness of ACS use in twin pregnancy
- Large sample size
- Internal pilot to assess recruitment rate and intra-cluster correlation co-efficient (ICC)
- Long term follow up only possible in a subset of participants within the timeframe of the trial

Manuscript Word Count: 3808

Introduction

The overall aim of STOPPIT-3 is to address the uncertainty regarding the effectiveness of Antenatal Corticosteroids (ACS) prior to a planned birth of twins in the late preterm and early term period. ACS are widely administered via intramuscular injection to women at risk of preterm birth (defined as birth less than 37 completed weeks gestation) to reduce morbidity and mortality in babies born too

For peer review only - http://bmjopen.bmj.com/site/about/guidelines.xhtml

For peer review only

early (1) and have been recommended since the 1990s. ACS are known to be most effective if birth occurs 24 to 48 hours following administration of the first dose, with little or no benefit seen if birth is seven days or more after administration (1).

Twin pregnancy is common and associated with adverse outcomes for the babies, accounting for ~3% of live births but ~15-20% of all neonatal care admissions (2). 2019 NICE guidance for twin pregnancy recommends planned birth at 37+0 weeks gestation in uncomplicated dichorionic (DC) twins (twins that have separate placentae), and planned birth at 36+0 weeks gestation in uncomplicated monochorionic (MC) twins (twins that share a placenta [~20% of twins])(3). Planned birth is by induction of labour (IOL) or caesarean section (CS). These slightly earlier, non-spontaneous births are at increased risk of respiratory morbidity and needing respiratory support requiring neonatal care admission.

There is, however, currently little evidence that ACS are as effective in twins, and similarly little evidence that ACS are effective in the late preterm and early term period which is the period that NICE recommend that twins are born (3). Evidence as to whether women having planned birth of twins should receive ACS is both conflicting and confusing, with practice known to be highly variable across the United Kingdom in this area. ACS are widely given to women with twin pregnancies having planned birth, despite recognition that ACS *may* have adverse effects on growth and neurodevelopment (4, 5). There is some evidence that ACS in singleton pregnancies in the late preterm period (34+0 – 36+6 weeks) and/or prior to planned CS at term (37+0 - 38+6 weeks gestation)(6), may have short term benefits reducing respiratory morbidity and neonatal care admission. This evidence is often extrapolated to twin pregnancy, however, there is almost no evidence showing benefits of ACS from women with twin pregnancies. Differences in the pharmacokinetics of ACS (7), and mechanisms of fetal maturation (which may be accelerated in twins)(8), may mean that ACS have different effectiveness at late preterm and early term gestations.

ACS are not devoid of harm. A large RCT of ACS in late preterm singletons demonstrated an increase in neonatal hypoglycaemia in the ACS group compared to placebo (number needed to harm 11)(9). ACS have well recognised detrimental effects on fetal growth (birthweight, length and head circumference) and conflicting results on neurodevelopment. Three studies (one RCT follow up and two longitudinal studies) have shown detrimental effects on neurodevelopment following ACS exposure (4, 5, 10) but a recently published prospective follow up study of the above RCTs of ACS in

For peer review only - http://bmjopen.bmj.com/site/about/guidelines.xhtml

late preterm singletons demonstrated no adverse effect of ACS on childhood neurodevelopment outcomes (11). The balance of risk and benefit needs to be determined for twin pregnancies. Reducing term (>37 weeks gestation) neonatal care admission is a UK national priority. It poses a high cost to the NHS and separation of mothers and babies is detrimental to maternal wellbeing, mother-infant bonding and breastfeeding (12). There is evidence that ACS reduce serious respiratory morbidity and neonatal unit admission but there is potential for short (e.g. hypoglycaemia) and long-term harms (e.g. neurodevelopment). Either currently a substantial number of babies miss a morbidity sparing treatment; or a substantial number receive a potentially harmful treatment unnecessarily as practice varies substantially across the UK. STOPPIT-3 will provide the evidence to address this uncertainty.

Methods

Design STOPPIT-3 is a multicentre double blind randomised placebo controlled trial to determine the clinical and cost effectiveness of ACS versus placebo in women with a viable twin pregnancy with planned birth between 35+0 and 38+6 weeks gestation. An internal pilot phase will take place to assess recruitment rates. A nested economics analysis will assess cost-effectiveness of ACS versus placebo. The primary objective is to test the hypothesis that ACS reduce neonatal morbidity including the need for respiratory support within 72 hours of birth. The secondary objectives are to determine the effect of ACS on severe respiratory morbidity, perinatal mortality, maternal outcomes including breastfeeding and infection and the cost-effectiveness of treatment with ACS compared to placebo. The effect of ACS compared to placebo on childhood cognitive and language development at the age of two will also be assessed in a subset of twins. The study opened for recruitment in August 2022 and recruitment will run until August 2025.

Health technology being assessed A single course of Dexamethasone Phosphate (24mg) given in two divided doses by intra-muscular (IM) injection to the thigh or buttock by appropriately qualified clinical or research staff 24 hours apart (+/- 4 hours). Two formulations of ACS, Dexamethasone and Betamethasone, are recommended in the UK. Dexamethasone has been chosen over Betamethasone as it does not need to be stored in a fridge, is cheaper and is more widely available Worldwide.

Population The target population is women with a confirmed viable twin pregnancy and planned birth between 35+0-38+6 weeks gestation. Women who are booked for their delivery at one of the

For peer review only

For peer review only - http://bmjopen.bmj.com/site/about/guidelines.xhtml

participating study sites and who appear to meet the study eligibility criteria will be invited to participate. Medical records of women pregnant with twins will be reviewed by the maternity care teams for individual recruitment potential into the trial. We anticipate that all eligible women expecting twins and attending for antenatal care in each of the sites will be invited to participate. Women who appear to fulfil the inclusion criteria for the trial will be approached by a member of maternity care team after confirmation of a viable twin pregnancy at an appropriate antenatal clinic or ultrasound visit, usually between 16-24 weeks gestation. Women will be provided with a written short trial summary at this time. Women will then be provided with a detailed patient information leaflet and consent form later in their pregnancy (between 32-36 weeks gestation, see supplementary material). The timings outlined for giving women trial information should be followed if possible, however flexibility for approaching women is permitted and deviation from the timelines set out here will not be recorded as a protocol deviation. If the woman waives this opportunity for early information but still wishes to participate, consent may be taken after a shorter time interval. Where possible the reason for an eligible woman being excluded or declining participation will be recorded, for input into trial metrics as per the CONSORT statement. (13)

Eligibility criteria The following inclusion criteria will apply at the screening assessment (all must apply):

Aged 16 years or older and able to provide electronic or written consent

Viable twin pregnancy (monochorionic or dichorionic) with a planned birth* scheduled between 35+0 and 38+6 weeks gestation including women who have a planned birth due to logistic reasons (e.g. availability of beds or staff), parental preference or other maternal or fetal indications.

Gestation established by scan at ≤16 weeks according to NICE guidelines and known chorionicity

≥ 24 hours* and < 7 days until planned birth

*Birth must be planned to take place at 35 or more weeks gestation, after induction of labour (IOL) or CS. At the point of randomisation there must be ≥ 24 hours until the planned CS or IOL date to

For peer review only - http://bmjopen.bmj.com/site/about/guidelines.xhtml

allow two doses of the study drug to be administered, at 24 hours (+/- 4 hours) apart prior to the planned birth.

The following exclusion criteria will apply:

Unable to give informed consent

Known or suspected major congenital fetal anomaly at the time of inclusion (defined as any structural or chromosomal anomaly that would influence management at or around birth or in the immediate postnatal period. Suspected isolated minor anomalies with lesser medical, functional or cosmetic consequences; or isolated limb abnormalities such as talipes can be included).

Diabetes (pre-existing or gestational) - Corticosteroid use may significantly disrupt glycaemic control in women with diabetes, with potential to 'unblind' treatment allocation and pose risk to these women. The effect of corticosteroids prior to planned CS in women with diabetes will be examined in other studies.

Receipt of ACS within the seven days prior to randomisation

Sensitivity, contraindication or intolerance to any of the ACS or any of its excipients

Chorionicity or gestational age are unknown

Other serious pregnancy morbidities which indicate either birth before 35 weeks or urgent birth within 24 hours

Outcomes

Primary outcome The primary outcome is need for respiratory support within 72 hours of birth. This outcome encompasses a range of levels of support consisting of one or more of the following: continuous positive airway pressure (CPAP); supplemental oxygen by high-flow nasal cannulae for at least 2 consecutive hours*;* need for supplemental oxygen by low flow nasal cannulae or incubator

For peer review only - http://bmjopen.bmj.com/site/about/guidelines.xhtml

oxygen for at least 4 continuous hours; mechanical ventilation; Extracorporeal Membrane Oxygenation (ECMO). Stillbirth or Neonatal death within 72 hours of birth will be included as competing events.

Secondary outcomes

Severe respiratory morbidity within 72 hours after birth (defined as one or more of the following: CPAP or high-flow nasal cannula for at least 12 continuous hours; Supplemental oxygen with a fraction of inspired oxygen of at least 0.30 for at least 24 continuous hours; Mechanical ventilation; ECMO; Stillbirth; Neonatal death within 72 hours of birth)

Any admission to neonatal care (i.e. admission for any reason and for any duration)

Neonatal care admission within 72 hours of birth for 48 hours or more or any Neonatal care admission (within 28 days of birth) or those requiring surfactant treatment or nitric oxide therapy

Apgar score at 5 minutes

Umbilical arterial cord pH

Umbilical arterial cord base excess

Newborn hypoglycaemia diagnosed within 48 hours of birth (defined as blood glucose of less than 2.0 mmol per litre).

Newborn neonatal jaundice (defined as those requiring treatment with phototherapy according to NICE threshold for gestation and postnatal age)

Birthweight centile

Head circumference at birth

All cause early onset sepsis within 72 hours of birth (defined as culture positive [pure growth from blood or CSF of a known bacterial pathogen] or culture negative [acute onset of illness with 3 or more predefined clinical signs])

Extended perinatal mortality (stillbirth or neonatal death up to 28 days)

For peer review only - http://bmjopen.bmj.com/site/about/guidelines.xhtml
For peer review only

Stillbirth (death *in utero*)

Neonatal death (death within 28 days of birth)

Exclusive breastmilk nutrition at discharge

Confirmed or suspected maternal postpartum infection during hospital admission (defined by a new prescription of antibiotics, confirmed systemic infection on culture, or endometritis as defined by the US Centers for Disease Control and Prevention)

Cost effectiveness of treatment with ACS compared to placebo

Childhood cognitive and language development at two years of age determined by the Parent Report of Children's Abilities-Revised (PARCA-R) score (14) (in the first 340 women recruited to the trial)

Consent and Baseline assessment

After the potential participant has had adequate time to consider involvement in the study, she will be contacted by a member of the trial team to ascertain interest in the trial. The consent, baseline assessment and randomisation for STOPPIT-3 are anticipated to be combined and conducted as a single visit before the planned birth and will wherever possible coincide with routine pre-admission appointments to help minimise additional visits. Written informed consent will be taken by a member of the maternity care team. Consent should be provided within 7 days of randomisation/IMP administration. The original signed consent form will be stored in the Investigator Site File, with a copy given to the woman and a copy added to the medical notes. The women's demographics, medical history, obstetric history, current pregnancy information and inclusion /exclusion criteria will be collected and entered on the eCRF by a member of the trial team. The inclusion/ exclusion criteria will be further assessed by a doctor (delegated by the PI) and they will complete and sign the eligibility form confirming the woman meets the study criteria to participate and is suitable for randomisation. A letter will be sent to the registered GP to inform them of the woman's participation in the trial.

Randomisation

Randomisation to ACS or placebo will be performed immediately prior to administration, 24 hours to 120 hours before the planned birth. Randomisation is performed using a web-based randomisation system managed by ECTU via a web portal. Users will be assigned a unique study identifier and will

For peer review only - http://bmjopen.bmj.com/site/about/guidelines.xhtml
For peer review only

be required to enter minimal patient details prior to randomisation. As this is a large trial (1552 women), group imbalances are unlikely therefore a simple allocation sequence with no minimisation criteria will be used. Study participants, trial investigators and medical staff providing care will remain blinded to treatment allocation. The randomisation process will assign each participant with a study drug treatment pack number and the first dose of IMP should be given immediately following randomisation with the second dose administered 24 hours (+/- 4 hours) after the first dose. Participants will be allocated to receive either:

1. Corticosteroid group – two doses of 12mg dexamethasone by IM injection 24 hours (+/- 4 hours) apart.

2. Placebo group – two doses of matching placebo (sodium chloride 0.9%) by IM injection 24 hours (+/- 4 hours) apart.

Data collection and management

Birth and neonatal information will be extracted from the woman's +/-babies' medical notes and information recorded in the eCRF by a member of the maternity research team. Trial data will be collected by members of the maternity care team delegated by the PI. A unique trial identifier will be allocated to each participating woman at randomisation and this unique number will be used for data collection within the trial. Identifiers will be stored in separate tables from the main data tables within the trial database and only delegated members of the team will be granted access to these tables (see Data Sharing Plan, supplementary material).

Long term Follow up Assessments

The first 340 STOPPIT-3 participants recruited will be asked to complete the PARCA-R questionnaire (on-line or paper copy) at 2 years (to assess the cognitive and language development).

Statistical analysis and sample size

The statistical analysis will be according to the intention to treat principle (i.e. all participants will remain in their allocated group for analysis). Statistical significance will be at the 5% level with corresponding 95% confidence intervals (CI) presented. Randomised groups will be described at baseline and follow-up using mean (SD), median (IQR) and counts (with percentages) as appropriate.

For peer review only - http://bmjopen.bmj.com/site/about/guidelines.xhtml

For the primary outcome (respiratory support within 72 hours of birth) the odds ratio (and 95% CI) for the treatment effect of ACS will be estimated adjusting for mode of delivery, treatment centre (if appropriate) and chorionicity with logistic regression. To account for the clustering effect within twin pairs, a random effects logistic regression model will be used by fitting pregnant woman as a random effect.

Continuous secondary outcomes will be analysed using linear regression, and binary categorical secondary outcomes will be analysed using logistic regression as per the primary outcome. Secondary outcomes with more than two categories will be analysed using multinomial logistic regression.

Subgroup analyses, for example by sex of twins, chorionicity and presence of maternal co-morbidity (e.g. hypertension) will be considered.

No interim analyses are planned other than re-estimation of the intraclass correlation (ICC, assumed to be 0.3) following the internal pilot. This will be done by estimating the 95% CI (without, and possibly with, adjustment for covariates) as per the event rate around the observed ICC at 200 women with complete data, and if this 95% CI does not contain 0.3 corrective action will be taken.

We plan to recruit 1552 women randomised at 1:1 to ACS or placebo prior to planned birth. We will have 90% power at the 5% significance level to detect a relative difference in the neonatal primary outcome of respiratory support within 72 hours of birth between the groups of 33% (absolute difference of 4%) assuming an event rate of 12% in the placebo group and an ICC of 0.3, assuming 1% of missing data for the primary outcome.

Health economic analysis

The primary within trial analysis will be a cost-effectiveness analysis (CEA) which will estimate the incremental cost per reduction in respiratory support (initiated within 72 hours after birth, i.e. the study primary outcome), with the time horizon spanning from birth to child hospital discharge or 28 days, whichever is sooner.

The costs of the intervention will be calculated as the daily cost of ACS medication and the associated administration costs. Hospital attendances required to administer ACS will be included. The direct medical costs post birth will be calculated based on resource utilisation accruing for the care of new born (after birth respiratory treatment; admission to neonatal care) and women (type of delivery,

For peer review only - http://bmjopen.bmj.com/site/about/guidelines.xhtml

inpatient stays; hospital transfers etc.) including adverse events. Resource utilisation for woman and child will be collected from the clinical hospital records up to 28 days post birth.

The mean cost and mean outcome associated with the intervention and the control arm will be estimated using generalised linear model (GLM), which will tackle non-normality of data, adjusting for relevant covariates (e.g. type of delivery; monochorionic/dichorionic twins), and adjusting for women-level clustering, in line with the statistical analysis.

If evidence of differences between the treatment arms in terms of effectiveness, costs or cost-effectiveness are found in the trial, a decision analytic model will be developed to explore the cost-effectiveness of ACS administration over a medium (2 year) and longer term (lifetime) horizon. The medium term analysis will utilise data from the final trial follow-up period in childhood (e.g. PARCA-R, any medical records available etc), to account for costs and consequences which are associated with ACS treatment over the neonatal period (hypoglycaemia; neonatal health) and childhood (mortality; cognitive development metabolic illness).

Internal Pilot

There will be an internal pilot phase over the first 10 months of the trial when we aim to recruit 159 women and have 36 sites open. There is a clear stop/go traffic light criterion for trial progression beyond the internal pilot.

Co-enrolment

Co-enrolment in STOPPIT-3 and another non-interventional research study (for example, sample only or questionnaire studies) is permitted and this does not require any formal written documentation. This includes the related STOPPIT-3 mechanistic study (STOPPIT-M) sponsored by the NIHR Efficacy and Mechanism Evaluation (EME) programme (reference NIHR133388).

Co-enrolment in STOPPIT-3 and another CTIMP or interventional non-CTIMP (for example, diagnostic, device or surgical interventions) are permitted provided an assessment on the safety of study participants, interventions involved, participant burden and the potential impact on the study endpoints have been considered. This assessment will be performed and documented in line with the Sponsor policy on co-enrolment.

For peer review only - http://bmjopen.bmj.com/site/about/guidelines.xhtml

Ineligible and non-recruited participants

Women who consent to participate in the study, but who spontaneously give birth or undergo IOL or CS prior to randomisation will not be eligible for randomisation. Such women who did consent to participate will be withdrawn but will remain on the eCRF system and reported in recruitment metrics as ineligible post consent. No delivery outcomes will be collected and they will continue receiving standard care under the management of a clinician, as per current guidelines. The woman's care will not be affected due to non-trial participation. Randomisation and IMP administration should be performed contiguously to minimise the chance of spontaneous labour or delivery between randomisation and IMP administration.

Unblinding

Breaking of the study blind will only be performed where knowledge of the treatment is essential for the clinical management of the woman or neonate. Unblinding is managed by the central Edinburgh team.

Withdrawal of study participants

Patients are free to withdraw at any point or can be withdrawn by the investigator. The primary reason for withdrawal will be recorded in the patient's eCRF and medical record.

Trial management and oversight

The multi-site trial will be coordinated by a Project Management Group consisting of the grant holders and the Trial Management Team within the Edinburgh Clinical Trials Unit (ECTU). A Trial Steering Committee (TSC) will be established to oversee the conduct and progress of the trial. An independent data monitoring committee (DMC) will be established to oversee the safety of participants in the trial.

Patient and Public Involvement (PPI)

The study was designed in response to a recent Global priority setting partnership of 1000 parents of twins who identified ten research priorities for future health of multiples and their families. Two of the top ten priorities will be addressed within STOPPIT-3 (i) How can we reduce multiples' (the babies) admission to the NNU and can we reduce their length of stay in the NNU and (ii) what are the

For peer review only - http://bmjopen.bmj.com/site/about/guidelines.xhtml

short and long-term outcomes in multiple pregnancies and are these outcomes affected by antenatal events and medical interventions?

The study has been co-designed with two charities who represent parents with twins, the Twins Trust and the Elizabeth Bryan Multiple Birth Centre (formerly the Multiple Births Foundation). We consulted parents, through both charities at the grant submission stage and also at the protocol stage specifically on study design, the primary outcome and effect size, secondary outcomes and recruitment strategies.

Patients and the public are also involved in the TSC for this study with two individual patients as well as involvement of the co-applicants from the Twins Trust and the Elizabeth Bryan Multiple Birth Centre. A virtual parent advisory group (PAG) has been set up to review patient facing materials and advise on dissemination plans. Individual study participants will be sent a summary of the study findings when the main study is published.

Ethics and dissemination

The study will be conducted in accordance with the principles of the International Conference on Harmonisation Tripartite Guideline for Good Clinical Practice (ICH GCP). The results of the study, together with other mandated information, will be uploaded to the European clinical trials database within 1 year of the end of the study. Summaries of results will also be made available to Investigators for dissemination within their clinics (where appropriate and according to their discretion). The results will be disseminated via publication in peer-reviewed journals and conference presentation and will also be communicated to the public via links with charity partners and social media.

Footnotes

Authors' contributions: SRM, SJS, MD, KB, JEN, JN, RMR, JPB, KL, AK, DB, KR, NF and JD developed the protocol. SRM, JT, RCT and SJS drafted the protocol. MD, KB, JEN, JN, RMR, JPB, KL, AK, DB, KR, NF, CK, and JD reviewed and commented on the protocol. SRM and JT contributed equally to the writing of this paper.

For peer review only - http://bmjopen.bmj.com/site/about/guidelines.xhtml

Funding Statement: This work was supported by the National Institute of Healthcare Research Health Technology Assessment grant number: NIHR131352

Competing interests: nil

Ethics approval: West Midlands - Edgbaston Research Ethics Committee REC reference: 22/WM/0018

Peer review: This study was extensively peer reviewed as part of the process of gaining grant funding from the NIHR HTA.

For peer review only

References

1. Roberts D, Dalziel S. Antenatal corticosteroids for accelerating fetal lung maturation for women at risk of preterm birth. Cochrane Database Syst Rev. 2006(3):Cd004454.

2. Harrison W, Goodman D. Epidemiologic Trends in Neonatal Intensive Care, 2007-2012. JAMA pediatrics. 2015;169(9):855-62.

3. National Institute for Health and Care Excellence. Twin and triplet pregnancy. 2019;NICE guideline NG137.

4. Stutchfield PR, Whitaker R, Gliddon AE, Hobson L, Kotecha S, Doull IJM. Behavioural, educational and respiratory outcomes of antenatal betamethasone for term caesarean section (ASTECS trial). Archives of disease in childhood Fetal and neonatal edition. 2013;98(3):F195-200.

5. Räikkönen K, Gissler M, Kajantie E. Associations Between Maternal Antenatal Corticosteroid Treatment and Mental and Behavioral Disorders in Children. JAMA : the journal of the American Medical Association. 2020;323(19):1924-33.

6. Saccone G, Berghella V. Antenatal corticosteroids for maturity of term or near term fetuses: systematic review and meta-analysis of randomized controlled trials. BMJ (Online). 2016;355:i5044-i.

7. Kemp MW, Newnham JP, Challis JG, Jobe AH, Stock SJ. The clinical use of corticosteroids in pregnancy. Human reproduction update. 2016;22(2):240-59.

For peer review only - http://bmjopen.bmj.com/site/about/guidelines.xhtml

8. Murray SR, Stock SJ, Cowan S, Cooper ES, Norman JE. Spontaneous preterm birth prevention in multiple pregnancy. Obstet Gynaecol, 2018;20 (1): 57-63.

9. Gyamfi-Bannerman C, Thom EA, Blackwell SC, Tita ATN, Reddy UM, Saade GR, et al. Antenatal Betamethasone for Women at Risk for Late Preterm Delivery. The New England journal of medicine. 2016;374(14):1311-20.

10. Rodriguez A, Wang Y, Khan AA, Cartwright R, Gissler M, Järvelin M-R. Antenatal corticosteroid therapy (ACT) and size at birth: A population-based analysis using the Finnish medical birth register. PLoS medicine. 2019;16(2):e1002746-e.

11. Gyamfi-Bannerman. Neurodevelopmental outcomes after late preterm antenatal corticosteroids: the alps follow-up study. AJOG. 2023 S764-S765.

12. Improvement N. Reducing harm leading to avoidable admission of full-term babies to neonatal units. London: NHS Improvement. 2017.

13. Schulz KF, Altman DG, Moher D. CONSORT 2010 Statement: updated guidelines for reporting parallel group randomised trials. BMJ. 2010;340:c332.

14. Johnson S BV, Linsell L, Brocklehurst P, Marlow N, Wolke D, Manktelow B. Parent Report of Children's Abilities – Revised (PARCA-R). Technical and Interpretive Manual The University of Leicester2019 [Available from: https://www2.le.ac.uk/partnership/parca-r/questionnaire).

For peer review only - http://bmjopen.bmj.com/site/about/guidelines.xhtml

Academic and Clinical Central Office for Research and Development

The STOPPIT Study Data Sharing Plan

STOPPIT-3

STOPPIT-M

**Study Title**	STOPPIT-3: A Randomised Placebo-Controlled Trial of Antenatal Corticosteroids for Planned Birth in Twins:<br><br>STOPPIT-M: Infant hypothalamic-pituitary-adrenal axis responses following antenatal corticosteroids and perinatal outcomes: a mechanism of action of health intervention study
**Chief Investigator**	STOPPIT-3: Professor Sarah Stock, Co-CI Dr. Sarah Murray<br>STOPPIT-M: Professor Rebecca Reynolds
**ISRCTN Number**	59959611
**Sponsor**	University of Edinburgh & NHS Lothian
**Version**	v1.0 11 October 2023

Table of Contents

1 Introduction 2
2 Data type 2
2.1 Type of scientific data expected to be generated in the trial 2
2.2 Dataset responsibility 2
2.3 Other associated documentation 3
2.3.1 Protocol 3
2.3.2 Statistical Analysis Plan / Health Economics Analysis Plan 3

For peer review only - http://bmjopen.bmj.com/site/about/guidelines.xhtml

accord
Academic and Clinical Central Office for Research and Development

2.3.3 Publication material ... 3
3 Data preservation, access, and timelines ... 3
3.1 Where will scientific data be archived ... 3
3.2 Archiving timelines ... 3
3.3 Data Access ... 3
3.3.1 Application Type ... 3
3.3.2 Application Process ... 4
4 Access, Distribution, or Reuse Considerations ... 4
4.1 Factors affecting subsequent access ... 4
4.2 What form will the sharable dataset take ... 4
4.3 Restrictions on data sharing ... 4
4.4 Protections for privacy, rights, and confidentiality of human research participants ... 4
4.5 Process of de-identification/anonymisation of the data ... 4
5 Oversight of Data Management and Sharing ... 4
6 Acknowledgement in output ... 4
7 References ... 4

1 Introduction

The STOPPIT 3 and STOPPIT M study data is held within Edinburgh Clinical Trials Unit (ECTU). The STOPPIT 3 data sharing plan therefore aligns with the ECTU Central Office SOP ECTU_OP_15: Data Access Request and Application Management SOP (Version 2.0; 11 Oct 2021) **[1].**

This data sharing plan is also in line with the STOPPIT 3 & STOPPIT M Publication policy **[2]**.

This data sharing plan has been approved by the Chief Investigator and the STOPPIT-3 trial statistician in Edinburgh Clinical Trials Unit (ECTU).

2 Data type

2.1 Type of scientific data expected to be generated in the trial

The data to be shared includes both meta data including the study protocol, case report forms and data dictionaries, and research participant data.

2.2 Dataset responsibility

The study team are responsible for the sharing of datasets arising from the STOPPIT 3 & STOPPIT M study. The Sponsor (University of Edinburgh and NHS Lothian) are joint data controllers for the study.

For peer review only - http://bmjopen.bmj.com/site/about/guidelines.xhtml

2.3 Other associated documentation

2.3.1 Protocol

The STOPPIT 3 & STOPPIT M study was registered on the ISRCTN clinical trials registry (https://www.isrctn.com/ISRCTNISRCTN599596111) before the participant recruitment commenced. The protocol will also be published in an open access journal.

2.3.2 Statistical Analysis Plan / Health Economics Analysis Plan

The Statistical Analysis Plan (SAP) will be available on request.

The Health Economics Analysis Plan (HEAP) will also be available on request.

2.3.3 Publication material

The STOPPIT 3 and STOPPIT M publication and dissemination policy describes how trial outputs will be managed, reviewed and disseminated. This policy follows ICMJE criteria for authorship. It outlines the requirements for research outputs from the study and any additional requirements for reporting and disseminating results. It is expected that study findings will be published as soon as possible, in a peer-reviewed open access journal or platform. The final report for NIHR HTA Journals Library will be submitted within 24 months of the end of the study (as defined in the protocol).

3 Data preservation, access, and timelines

3.1 Where will scientific data be archived

Once the study is closed and the statistical analysis has been completed, all study data will be stored on a suitable secure server in the Edinburgh Clinical Trials Unit. Study data will be available on request from the ECTU data sharing team (see section 3.3).

3.2 Archiving timelines

Study data and metadata will be kept for a minimum of 25 years from the protocol defined end of study point. When the minimum retention period has elapsed, study documentation will not be destroyed without permission from the sponsor.

3.3 Data Access

3.3.1 Application Type

Applications for access to study data which are received from an external source, out with ECTU, are subject to review by the ECTU Data Sharing team. There are two categories of data access request. A Data Access Request Application Type A is made when the study is currently recruiting participants, closed to recruitment with participants in follow-up or when the study is closed (all recruitment and follow-up completed) but the main statistical analysis is not yet complete. This should be made using form Data Access Application Form Type A (OP-F02).

A Data Access Request Application Type B is made when the study is closed, and the statistical analysis has been completed. This should be made using form Data Access Application Form Type B (OP-F03)

For peer review only - http://bmjopen.bmj.com/site/about/guidelines.xhtml

3.3.2 Application Process

In the first instance, email requests for study data should be made via email to: ECTUdatashare@ed.ac.uk.

Further details about this process are available in ECTU Central Office SOP ECTU_OP_15 **[1].**

4 Access, Distribution, or Reuse Considerations

4.1 Factors affecting subsequent access

If the application is approved, the review panel will also consider the method of access and whether any additional agreements will be required prior to the access being granted. It may be necessary to further consult with external colleagues (e.g. contracts) at this stage.

4.2 What form will the sharable dataset take

The sharable dataset will include the statistical analysis dataset and the health economic analysis dataset. In general, we will only share the statistical analysis dataset and the health economics data set, but consideration will be given to sharing source dataset tables upon request.

4.3 Restrictions on data sharing

If the study results have not yet been published, it may be appropriate to embargo any data access requests until post-publication to ensure the results are not undermined.

4.4 Protections for privacy, rights, and confidentiality of human research participants

All shared data will be de-identified prior to release and in accordance with permissions listed in the STOPPIT 3 & STOPPIT M protocol, ethical approvals, and Patient Information Sheet Consent Form (PISCF)

4.5 Process of de-identification/anonymisation of the data

Suitably qualified personnel will de-identify the data prior to release, if his has not already been done.

5 Oversight of Data Management and Sharing

ECTU will retain oversight of data management, and of sharing processes for requests that come to ECTU, involving data held by ECTU.

6 Acknowledgement in output

Secondary users of data should follow the STOPPIT 3 and STOPPIT M Publication policy for acknowledgments and authorship **[2]**.

7 References

For peer review only - http://bmjopen.bmj.com/site/about/guidelines.xhtml

Academic and Clinical Central Office for Research and Development

1. ECTU Central Office SOP ECTU_OP_15: Data Access Request and Application Management SOP; Version 2.0; 11 Oct 2021; available at https://www.ed.ac.uk/sites/default/files/atoms/files/ectu_sop_op_15_data_access_request_and_application_management_v2.0.pdf (accessed 11th November 2022).

2. STOPPIT-3 & M Publication Policy; Version 1.0; 17 Jan 2022; available from the STOPPIT trial management team at stoppit.trial@ed.ac.uk

For peer review only

For peer review only - http://bmjopen.bmj.com/site/about/guidelines.xhtml

INSERT LOCAL DETAILS HERE

Participant Information Sheet

A Randomised Placebo-Controlled Trial of Antenatal Corticosteroids for Planned Birth in Twins: STOPPIT-3

You are invited to take part in a research trial. To help you decide whether or not to take part, it is important for you to understand why the research is being done and what it will involve. Please take time to read the following information carefully. Talk to others about the trial if you wish. Contact us if there is anything that is not clear, or if you would like more information. Take time to decide whether or not you wish to take part.

Why have I been invited to take part?

You have been asked to take part as you are pregnant with twins.

What is the purpose of the trial?

This trial aims to find out if the drug antenatal corticosteroids (ACS) given to women with a twin pregnancy prior to a planned birth of twins after 35 weeks of pregnancy reduces breathing difficulties in the twin babies.

Antenatal Corticosteroids (ACS) help to mature babies' lungs and may reduce breathing difficulties and the need for high levels of respiratory support. They are routinely used in singleton pregnancies which deliver early, but the use of ACS in twin births has not been studied in detail and so it is not clear if they will work in twin pregnancies. Because of the lack of evidence, there is currently no guidance on giving ACS in twin pregnancies, so whether or not women pregnant with twins receive steroids as part of routine care varies depending on their hospital. ACS may also have some unwanted side effects such as lowering babies' blood sugars, affecting babies' growth and possibly affecting the babies' brain development. We need to be certain about the benefits and risks of giving ACS before all women with twin pregnancy in the UK are offered a course of ACS prior to a planned birth.

Twin pregnancies are monitored more closely as they have a higher risk of complications than a singleton pregnancy, and there is a greater chance of the babies being born before 37 weeks of pregnancy. Twin births account for about 3% of live births but around 15-20% of admissions to the neonatal unit.

Current guidance recommends that twins who share a placenta (monochorionic twins) should be born from 36 weeks of pregnancy if there are no medical problems requiring earlier birth, whilst twins with a placenta each (dichorionic twins) should be born from 37 weeks of pregnancy, as evidence shows this is safer than delivering later on in the pregnancy. Being born slightly early means that twins are at higher risk of admission to neonatal units for support with their breathing, which separates mothers and babies at a crucial time.

FUNDED BY NIHR | National Institute for Health Research

This study is funded by the National Institute for Health Research (NIHR) Health Technology Assessment (HTA) programme (NIHR131352). The views expressed are those of the author(s) and not necessarily those of the NIHR or the Department of Health and Social Care.

For peer review only - http://bmjopen.bmj.com/site/about/guidelines.xhtml

INSERT LOCAL DETAILS HERE

We are performing this trial in NHS Centres throughout the UK. Women with a twin pregnancy who have a planned birth after 35 weeks of pregnancy are invited to participate in the trial. Women who agree to take part in the trial will be treated with either ACS or a placebo (dummy drug). A placebo, or dummy drug, is an inactive substance which seems to be a "real medical treatment". In this trial the placebo is a sodium chloride solution which will look identical to the ACS injections. These will be administered by injection prior to the planned birth.

We will compare the two groups to see if there are differences in the need for extra healthcare support after birth. If we find that the use of ACS improves health in twin babies, it could be used in the NHS straight away.

We need around 1,550 women to participate in the trial to be able to see if ACS works in twins.

Do I have to take part?

No, it is up to you to decide whether or not to take part. If you do decide to take part, you will be given this information sheet to keep and be asked to sign a consent form. If you decide to take part, you are still free to withdraw at any time and without giving a reason. Deciding not to take part or withdrawing from the trial will not affect the healthcare that you receive, or your legal rights.

What will happen if I take part?

An outline of the trial is given below. Where possible we will combine any additional visits needed for the trial with your routine antenatal appointments to avoid too many extra appointments However, combining the trial visit with routine visits may mean a longer visit overall. Some of the assessments may be carried out virtually before the visit using a remote secure system.

Giving consent to take part

The maternity care team will review your maternity notes and determine whether you are eligible to take part in the trial. If you are eligible, you will be asked if you would like to participate by a member of the maternity team, and this will usually be during one of your routine antenatal appointments. If you have verbally agreed to participate you will be invited to attend hospital 24 -120 hours (1-5 days) hours prior to admission for planned birth, when the ACS or placebo will be administered. You will be asked to sign a consent form before the trial drug is administered, and you will be given a copy of the signed consent form to keep for your records.

If you consented to take part but your baby is born before 35 weeks' gestation, or you are induced before your planned delivery date, you will not be eligible to proceed in the trial. Whether or not you receive ACS in this case will be decided by your doctor on an individual basis.

Trial data collection

If you consent to take part, a member of the research team will collect some information about you, including:

- medical history, including current medication
- obstetric history (previous pregnancies/births)

FUNDED BY NIHR | National Institute for Health Research

This study is funded by the National Institute for Health Research (NIHR) Health Technology Assessment (HTA) programme (NIHR131352). The views expressed are those of the author(s) and not necessarily those of the NIHR or the Department of Health and Social Care.

For peer review only - http://bmjopen.bmj.com/site/about/guidelines.xhtml

INSERT LOCAL DETAILS HERE

- current pregnancy information

This will be entered on to the trial database by a member of the research team.

Randomisation

Sometimes we don't know which treatment is best. To find out, we need to make comparisons between different treatments. We do this by putting people into groups and giving each group a different treatment; the results are then compared to see if one is better. To try to make sure the groups are the same to start with, each participant is put into a group by chance (randomly). This is called randomisation. The results are then compared.

In STOPPIT-3 you will be randomised to one of two groups. There is approximately a 50:50 chance that you will be randomised into either group.

1. Corticosteroid Group: Two separate doses of ACS (Dexamethasone) by intramuscular injection (either to the thigh or buttock)

OR

Placebo Group: Two separate doses of visually matching placebo (Sodium Chloride, also known as saline) by intramuscular injection (either to the thigh or buttock).

The trial is a double blind trial, and so neither you nor your doctor/medical team will know which treatment group you are in (although, if your doctor needs to find out s/he can do so). Everyone involved in the trial - women, medical professionals caring for women and trial investigators - will remain blinded to treatment allocation until the trial is completed.

You will be given a study card with contact information in case you have any questions. This card should be used if clinical staff need to know which treatment you received in an emergency. This process is called 'unblinding' and the treatment information needed can be obtained by the clinical team and the central trial team who will use the approved study database to obtain the information required. You should carry this study card with you while in the trial.

Your GP will be informed, by letter, of your participation in STOPPIT-3.

We hope to establish if there are any effects of ACS for the mothers. Women have told us the outcomes that are important to them, and so we will be looking at rates of infection after birth and any impact on breastfeeding. We will also look at any complications in babies. Information about the health of you and your baby in the period after birth will be taken from your medical records. We will collect data from you/your babies' medical record until discharge or 28 days postnatal (whichever is sooner).

We will ask parents to complete a questionnaire (online or by post if you prefer) called a PARCA-R (Parent Report of Children's Abilities-Revised). This questionnaire will be used to assess children's development. This will be done when your babies are 2 years old, which is when the questionnaire is designed to be used and is a good time to get an indication of any long-term positive effects of ACS on babies' health. Before sending the questionnaire to you we will ask the maternity care team to check that the details you have given us are still up to date.

If any new information about the drug we are studying becomes available during the course of the trial, all women will be informed by their preferred method of communication.

FUNDED BY NIHR | National Institute for Health Research

This study is funded by the National Institute for Health Research (NIHR) Health Technology Assessment (HTA) programme (NIHR131352). The views expressed are those of the author(s) and not necessarily those of the NIHR or the Department of Health and Social Care.

For peer review only - http://bmjopen.bmj.com/site/about/guidelines.xhtml

INSERT LOCAL DETAILS HERE

If you decide to take part in STOPPIT-3 you may still be eligible to take part in other research studies involving medicines and this will be checked by the STOPPIT-3 team. You will also be able to take part in other types of research, for example studies where you are asked to complete a questionnaire, or studies that are looking at the data collected when you are treated in hospital.

What are the possible benefits of taking part?

We don't know if you and your baby will directly benefit from taking part in this trial. Information we obtain from your participation in the study may help inform on the future healthcare of other patients. Taking part will help create much needed evidence on the use of ACS prior to a planned birth of twins, which will help women and babies in future.

What are the possible disadvantages of taking part?

You may be required to spend some extra time at a routine antenatal visit. If you do choose to take part in the study you may also be required to spend extra time at the hospital when you attend the planned pre-birth appointment. This is because we will need to record extra information specifically for the trial, for example, it will take time to discuss the trial with you, and to take consent to participate. This is additional to what is normally discussed at antenatal appointments and so will take a little extra time.

Where possible any additional visits required for the trial will be combined with routine antenatal appointments. Therefore you will not receive any recompense for taking part in the trial; this includes things like travel expenses. However you will receive a £20 high street shopping voucher for completing the follow-up questionnaire as recognition for your time and input.

There are very few recognised immediate side effects of a short course of ACS as used in this study. ACS are routinely used in pregnancy when women are at risk of preterm birth with very low rates of side effects. Allergic reactions to ACS are extremely rare. Headaches and short-term sleep disturbance have been reported after ACS but not confirmed.

What if there are any problems?

If you have a concern about any aspect of this trial please contact <insert name and contact details here> who will do their best to answer your questions.

In the unlikely event that something goes wrong, and you are affected during the research and this is due to someone's negligence, then you may have grounds for a legal action for compensation against your local hospital or the trial sponsors: University of Edinburgh/NHS Lothian, but you may have to pay your legal costs. The normal National Health Service complaints mechanisms will still be available to you (if appropriate).

What will happen if I don't want to carry on with the trial?

You can withdraw from the trial at any point, without giving a reason; this would not affect your clinical care. We will keep information about you that we already have.

FUNDED BY NIHR | National Institute for Health Research

This study is funded by the National Institute for Health Research (NIHR) Health Technology Assessment (HTA) programme (NIHR131352). The views expressed are those of the author(s) and not necessarily those of the NIHR or the Department of Health and Social Care.

For peer review only - http://bmjopen.bmj.com/site/about/guidelines.xhtml

INSERT LOCAL DETAILS HERE

What happens when the trial is finished?

We will write a clinical trial report, which may be used for publication and presentation at scientific meetings. All information in this report will be anonymised. These results will be uploaded to a publically accessible database within a year of the trial ending.

All trial data will be kept for at least 25 years from the end of the trial.

Will my taking part be kept confidential?

All the information we collect during the course of the research will be kept confidential and there are strict laws which safeguard your privacy at every stage.

How will we use information about you?

We will need to use information from you and your babies from your hospital notes for this research project.

Any details we have about you will be kept securely, with access restricted on a secure bespoke database managed by the University of Edinburgh Clinical Trials Unit (ECTU). This information will be used to contact you about the trial by doctors or researchers running this trial. With your consent we will collect the following personal information:

- Name, ethnicity, Dates of Birth and Hospital number (NHS or CHI) for both you and your babies. The NHS number or Community Health Index number (in Scotland) is used for health care purposes and it uniquely identifies a person.
- Address (postal and email) and contact details: this is so we can continue to follow up your babies and contact you at the end of the trial with information about the results.
- If you are asked (and agree) to sign the consent form electronically the IP address from the computer you use to sign the form will be collected

Personal information collected will be retained (with your consent) for use in future studies into the long term outcomes of ACS. It is necessary to keep personal information for you and your babies to link trial data (treatment group) to long term outcomes (NHS records/school records). Any future studies would require separate governance approvals.

We will inform your GP that you are taking part, with your consent.

All the information we collect about you and your baby will be stored in a secure database and only the trial researchers will have access to this. When you are randomised, you will be allocated a unique trial number and individuals that do not need to know who you are will see only your trial number and not your personal information. All the information we collect during the course of the research will be kept confidential and there are strict laws which safeguard your privacy at every stage.

FUNDED BY NIHR | National Institute for Health Research

This study is funded by the National Institute for Health Research (NIHR) Health Technology Assessment (HTA) programme (NIHR131352). The views expressed are those of the author(s) and not necessarily those of the NIHR or the Department of Health and Social Care.

For peer review only - http://bmjopen.bmj.com/site/about/guidelines.xhtml

STOPPIT 3

INSERT LOCAL DETAILS HERE

We will keep all information about you safe and secure, and will not share any personal information held about you with any other organisation. However, individuals from the trial funder, regulatory authority or Sponsor organisation may review trial information and sections of you/your babies' medical notes to ensure that the trial is being done properly.

Once we have finished the trial, we will keep some of the data so we can check the results. We will write our reports in such a way that no-one can work out that you took part in the trial.

What are your choices about how your information is used?

You can stop being part of the trial at any time, without giving a reason, but we will keep the information about you that we already have. It is important that we keep the data collected up until the point at which you withdraw as it documents the care that you received and therefore forms part of your maternity care record.

If you choose to stop taking part in the trial, we will ask you if we can continue collecting information about your health and your babies' health from your hospital notes. If you do not want this to happen, tell us and we will stop. We will discuss this with you but if you decide not to participate further the research team will collect this information from your notes unless you tell them (or another member of the clinical team) that you do not agree to this. We think this is important to collect this information, so that we can find out if giving ACS is beneficial.

We need to manage your records in specific ways for the research to be reliable. This means that we won't be able to let you see or change the data we hold about you. Research could go wrong if data is removed or changed.

Future research studies organised by other Universities may also investigate more about the effects of ACS in twins. With your permission, we would like to share _anonymised_ information collected during this trial with other researchers running similar studies in the future. If you do not want your anonymised information to be shared with other organisations for future research you must make this clear in the attached consent form

Some studies may ask to use data that identifies you. If this is the case, a member of the trial team from Edinburgh University will contact you to discuss this and request permission. Identifiable data will be never be shared with another organisation without your consent.

Where can you find out more about how your information is used?
You can find out more about how we use your information here:
www.hra.nhs.uk/information-about-patients/

- our leaflet available from **www.hra.nhs.uk/patientdataandresearch**
- by asking one of the research team
- by sending an email to STOPPIT.Trial@ed.ac.uk or the Administrator: Lorraine Adamson, email: L.D.Adamson@ed.ac.uk

What will happen to the results of the trial?

The results of the trial will be published in research journals and presented at scientific meetings.

FUNDED BY NIHR | National Institute for Health Research

This study is funded by the National Institute for Health Research (NIHR) Health Technology Assessment (HTA) programme (NIHR131352). The views expressed are those of the author(s) and not necessarily those of the NIHR or the Department of Health and Social Care.

For peer review only - http://bmjopen.bmj.com/site/about/guidelines.xhtml

INSERT LOCAL DETAILS HERE

We will also update you (the participant) with a summary of the trial findings through our trial website and other social media platforms. We do not expect the trial results to be available until early 2026, and so we will update the website and social media sites with trial progress. All information used for trial updates and final results will be anonymous, and it will not be possible to identify individuals from any published material.

Who is organising and funding the research?

This trial is organised and sponsored by the University of Edinburgh and NHS Lothian.

The trial is being funded by the National Institute for Health Research (NIHR), Health Technology Assessment (HTA) Programme (Project: 131352). The views and opinions are those of the authors and do not necessarily reflect those of the HTA programme, NIHR or the Department of Health.

Who has reviewed the trial?

This trial has been reviewed and approved by the following bodies:

(1) Research Ethics Committee (REC). All research in the NHS is looked at by an independent group of people called a Research Ethics Committee. REC approval was obtained on 14/02/2022 (ref 22/WM/0018).

(2) Medicines and Healthcare Regulatory Agency (MHRA). The MHRA review and authorise all clinical research studies investigating the safety or efficacy of a drug. The MHRA approval was obtained on 15/02/2022 (ref CTA 01384/0268/001-0001).

(3) NHS Management Approval. Each hospital that takes part in a clinical trial must also review and approve the trial before their patients can be approached to take part. NHS Management Approval was obtained from (site name) on XX (ref)

(4) User groups/stakeholders. The trial protocol, information sheets and trial design have also been reviewed by relevant user groups and stakeholders. The Twins Trust and Multiple Births Foundation have provided input to the trial materials, and advised on various aspects of the management of the trial.

Researcher Contact Details

If you have any further questions about the trial, please contact <insert name> on <insert phone number> or email on: <insert email address>.

Independent Contact Details

If you would like to discuss this trial with someone independent of the trial, please contact TBC <insert contact details>.

Complaints

If you wish to make a complaint about the trial please contact:

Adapt depending on research site <insert contact details>

For NHS Lothian this is:
Patient Experience Team
2 – 4 Waterloo Place, Edinburgh, EH1 3EG
feedback@nhslothian.scot.nhs.uk Tel: 0131 536 3370

FUNDED BY NIHR | National Institute for Health Research

This study is funded by the National Institute for Health Research (NIHR) Health Technology Assessment (HTA) programme (NIHR131352). The views expressed are those of the author(s) and not necessarily those of the NIHR or the Department of Health and Social Care.

For peer review only - http://bmjopen.bmj.com/site/about/guidelines.xhtml

For peer review only

INSERT LOCAL DETAILS HERE

CONSENT FORM

A Randomised Placebo-Controlled Trial of Antenatal Corticosteroids for Planned Birth in Twins: **STOPPIT-3**

Please **initial** box

1. I confirm that I have read and understand the information sheet (STOPPIT-3 PIL Version 8.0, dated 27/06/2023) for the above trial. I have had the opportunity to consider the information, ask questions and have had these questions answered satisfactorily. ☐
2. I understand that my participation is voluntary and that I am free to withdraw at any time, without giving any reason and without my medical care and/or legal rights being affected. ☐
3. I give permission for the research team to access my and my babies medical records for the purposes of this research trial. ☐
4. I understand that relevant sections of my, and my babies', medical notes and data collected during the trial including my personal details may be looked at by individuals from the regulatory authorities and from the Sponsor(s) (NHS Lothian and the University of Edinburgh), from other NHS Boards or Trusts involved in the trial, where it is relevant to my taking part in this research. I give permission for these individuals to have access to my records. ☐
5. I give permission for my and my babies personal information (including name, address, date of birth, telephone number and consent form) to be passed to the University of Edinburgh Trials Unit for administration of the trial. ☐
6. I give permission for my Community Health Index (CHI) number/hospital number or NHS number to be collected and passed to the University of Edinburgh Clinical Trials Unit ☐
7. I agree to my General Practitioner being informed of my participation in the trial. ☐
8. I understand that data collected about me and my babies (related to the trial and personal) during the trial will be kept for 25 years, and might be contacted in the future about related research studies. ☐
9. I agree to my anonymised data being used in future studies. Yes ☐ No ☐
10. I understand that the information held and maintained by [enter site/hospital name] may be used to help contact me or provide information about my health status ☐
11. I agree to take part in the above trial. ☐

Name of Person Giving Consent	Date	Signature
Name of Person Receiving Consent	Date	Signature

1x original – into Site File; 1x copy – to Participant; 1x copy – into medical record

FUNDED BY NIHR | National Institute for Health Research

This study is funded by the National Institute for Health Research (NIHR) Health Technology Assessment (HTA) programme (NIHR131352). The views expressed are those of the author(s) and not necessarily those of the NIHR or the Department of Health and Social Care.

For peer review only - http://bmjopen.bmj.com/site/about/guidelines.xhtml

SPIRIT

STANDARD PROTOCOL ITEMS: RECOMMENDATIONS FOR INTERVENTIONAL TRIALS

SPIRIT 2013 Checklist: Recommended items to address in a clinical trial protocol and related documents*

Section/item	ItemNo	Description	Page No
**Administrative information**			
Title	1	Descriptive title identifying the study design, population, interventions, and, if applicable, trial acronym	1
Trial registration	2a	Trial identifier and registry name. If not yet registered, name of intended registry	3
	2b	All items from the World Health Organization Trial Registration Data Set	N/A
Protocol version	3	Date and version identifier	3
Funding	4	Sources and types of financial, material, and other support	15
Roles and responsibilities	5a	Names, affiliations, and roles of protocol contributors	1,2,14
	5b	Name and contact information for the trial sponsor	3
	5c	Role of study sponsor and funders, if any, in study design; collection, management, analysis, and interpretation of data; writing of the report; and the decision to submit the report for publication, including whether they will have ultimate authority over any of these activities	N/A
	5d	Composition, roles, and responsibilities of the coordinating centre, steering committee, endpoint adjudication committee, data management team, and other individuals or groups overseeing the trial, if applicable (see Item 21a for data monitoring committee)	13
**Introduction**			
Background and rationale	6a	Description of research question and justification for undertaking the trial, including summary of relevant studies (published and unpublished) examining benefits and harms for each intervention	3,4,5
	6b	Explanation for choice of comparators	5,6
Objectives	7	Specific objectives or hypotheses	7,8,9
Trial design	8	Description of trial design including type of trial (eg, parallel group, crossover, factorial, single group), allocation ratio, and framework (eg, superiority, equivalence, noninferiority, exploratory)	5
**Methods: Participants, interventions, and outcomes**			

For peer review only

Study setting	9	Description of study settings (eg, community clinic, academic hospital) and list of countries where data will be collected. Reference to where list of study sites can be obtained	5,6
Eligibility criteria	10	Inclusion and exclusion criteria for participants. If applicable, eligibility criteria for study centres and individuals who will perform the interventions (eg, surgeons, psychotherapists)	6,7
Interventions	11a	Interventions for each group with sufficient detail to allow replication, including how and when they will be administered	5
	11b	Criteria for discontinuing or modifying allocated interventions for a given trial participant (eg, drug dose change in response to harms, participant request, or improving/worsening disease)	13
	11c	Strategies to improve adherence to intervention protocols, and any procedures for monitoring adherence (eg, drug tablet return, laboratory tests)	N/A
	11d	Relevant concomitant care and interventions that are permitted or prohibited during the trial	12
Outcomes	12	Primary, secondary, and other outcomes, including the specific measurement variable (eg, systolic blood pressure), analysis metric (eg, change from baseline, final value, time to event), method of aggregation (eg, median, proportion), and time point for each outcome. Explanation of the clinical relevance of chosen efficacy and harm outcomes is strongly recommended	7,8,9,10,11,12
Participant timeline	13	Time schedule of enrolment, interventions (including any run-ins and washouts), assessments, and visits for participants. A schematic diagram is highly recommended (see Figure)	9,10
Sample size	14	Estimated number of participants needed to achieve study objectives and how it was determined, including clinical and statistical assumptions supporting any sample size calculations	10,11
Recruitment	15	Strategies for achieving adequate participant enrolment to reach target sample size	12,14

Methods: Assignment of interventions (for controlled trials)

Allocation:

Sequence generation	16a	Method of generating the allocation sequence (eg, computer-generated random numbers), and list of any factors for stratification. To reduce predictability of a random sequence, details of any planned restriction (eg, blocking) should be provided in a separate document that is unavailable to those who enrol participants or assign interventions	9,10

Allocation concealment mechanism	16b	Mechanism of implementing the allocation sequence (eg, central telephone; sequentially numbered, opaque, sealed envelopes), describing any steps to conceal the sequence until interventions are assigned	9,10
Implementation	16c	Who will generate the allocation sequence, who will enrol participants, and who will assign participants to interventions	9,10
Blinding (masking)	17a	Who will be blinded after assignment to interventions (eg, trial participants, care providers, outcome assessors, data analysts), and how	10
	17b	If blinded, circumstances under which unblinding is permissible, and procedure for revealing a participant's allocated intervention during the trial	13
**Methods: Data collection, management, and analysis**			
Data collection methods	18a	Plans for assessment and collection of outcome, baseline, and other trial data, including any related processes to promote data quality (eg, duplicate measurements, training of assessors) and a description of study instruments (eg, questionnaires, laboratory tests) along with their reliability and validity, if known. Reference to where data collection forms can be found, if not in the protocol	9,10
	18b	Plans to promote participant retention and complete follow-up, including list of any outcome data to be collected for participants who discontinue or deviate from intervention protocols	10,13
Data management	19	Plans for data entry, coding, security, and storage, including any related processes to promote data quality (eg, double data entry; range checks for data values). Reference to where details of data management procedures can be found, if not in the protocol	Supplementary material
Statistical methods	20a	Statistical methods for analysing primary and secondary outcomes. Reference to where other details of the statistical analysis plan can be found, if not in the protocol	10,11,12
	20b	Methods for any additional analyses (eg, subgroup and adjusted analyses)	10,11,12
	20c	Definition of analysis population relating to protocol non-adherence (eg, as randomised analysis), and any statistical methods to handle missing data (eg, multiple imputation)	10
**Methods: Monitoring**			
Data monitoring	21a	Composition of data monitoring committee (DMC); summary of its role and reporting structure; statement of whether it is independent from the sponsor and competing interests; and reference to where further details about its charter can be found, if not in the protocol. Alternatively, an explanation of why a DMC is not needed	13

For peer review only - http://bmjopen.bmj.com/site/about/guidelines.xhtml

	21b	Description of any interim analyses and stopping guidelines, including who will have access to these interim results and make the final decision to terminate the trial	12
Harms	22	Plans for collecting, assessing, reporting, and managing solicited and spontaneously reported adverse events and other unintended effects of trial interventions or trial conduct	13
Auditing	23	Frequency and procedures for auditing trial conduct, if any, and whether the process will be independent from investigators and the sponsor	NA
**Ethics and dissemination**			
Research ethics approval	24	Plans for seeking research ethics committee/institutional review board (REC/IRB) approval	14
Protocol amendments	25	Plans for communicating important protocol modifications (eg, changes to eligibility criteria, outcomes, analyses) to relevant parties (eg, investigators, REC/IRBs, trial participants, trial registries, journals, regulators)	11,14
Consent or assent	26a	Who will obtain informed consent or assent from potential trial participants or authorised surrogates, and how (see Item 32)	9
	26b	Additional consent provisions for collection and use of participant data and biological specimens in ancillary studies, if applicable	10
Confidentiality	27	How personal information about potential and enrolled participants will be collected, shared, and maintained in order to protect confidentiality before, during, and after the trial	10
Declaration of interests	28	Financial and other competing interests for principal investigators for the overall trial and each study site	15
Access to data	29	Statement of who will have access to the final trial dataset, and disclosure of contractual agreements that limit such access for investigators	DSP - Supplementary material
Ancillary and post-trial care	30	Provisions, if any, for ancillary and post-trial care, and for compensation to those who suffer harm from trial participation	NA
Dissemination policy	31a	Plans for investigators and sponsor to communicate trial results to participants, healthcare professionals, the public, and other relevant groups (eg, via publication, reporting in results databases, or other data sharing arrangements), including any publication restrictions	14
	31b	Authorship eligibility guidelines and any intended use of professional writers	14
	31c	Plans, if any, for granting public access to the full protocol, participant-level dataset, and statistical code	DSP - Supplementary material

For peer review

For peer review only - http://bmjopen.bmj.com/site/about/guidelines.xhtml

**Appendices**			
Informed consent materials	32	Model consent form and other related documentation given to participants and authorised surrogates	Supplementary materil
Biological specimens	33	Plans for collection, laboratory evaluation, and storage of biological specimens for genetic or molecular analysis in the current trial and for future use in ancillary studies, if applicable	NA

*It is strongly recommended that this checklist be read in conjunction with the SPIRIT 2013 Explanation & Elaboration for important clarification on the items. Amendments to the protocol should be tracked and dated. The SPIRIT checklist is copyrighted by the SPIRIT Group under the Creative Commons "Attribution-NonCommercial-NoDerivs 3.0 Unported" license.

For peer review only
