# Supplementary material for: Randomised placebo-controlled trial of antenatal corticosteroids for planned birth in twins (STOPPIT-3): study protocol
Source: BMJ Open. 2024 Jan 18;14(1):e078778. doi: 10.1136/bmjopen-2023-078778 (PMC10806667; doi:10.1136/bmjopen-2023-078778)
Supplement: Reviewer comments [file bmjopen-2023-078778.reviewer_comments.pdf]

PEER REVIEW HISTORY

BMJ Open publishes all reviews undertaken for accepted manuscripts. Reviewers are asked to complete a checklist review form (**http://bmjopen.bmj.com/site/about/resources/checklist.pdf**) and are provided with free text boxes to elaborate on their assessment. These free text comments are reproduced below.

ARTICLE DETAILS

**TITLE (PROVISIONAL)**	Study protocol: a randomised placebo-controlled trial of antenatal corticosteroids for planned birth in twins (STOPPIT-3)
**AUTHORS**	Murray, Sarah; Thompson, Jessica; Townsend, Rosie; Deidda, Manuela; Boyd, Kathleen; Norman, Jane; Norrie, John; Boardman, James; Luyt, Karen; Khalil, Asma; Bick, Debra; Reed, Keith; Denton, Jane; Fenwick, Natasha; Keerie, Catriona; Reynolds, Rebecca; Stock, Sarah

This article was not externally reviewed at BMJ Open. Protocols that have been independently assessed prior to submission to BMJ Open are usually fast-tracked to publication on the grounds that further substantial changes will not be possible. This independent assessment will usually be external, independent review for both a substantial grant award from a non-commercial or government funder and ethics approval.
